# A Geometry of Hamiltonian Mechanics

**DOI:** 10.3390/e28040379

**Published:** 2026-03-27

**Authors:** Gil Elgressy, Lawrence Horwitz

**Affiliations:** 1 Department of Physics, Bar Ilan University, Ramat Gan 52900, Israel; 2 School of Physics, Tel Aviv University, Ramat Aviv 69978, Israel; 3 Department of Physics, Ariel University, Ariel 44837, Israel

**Keywords:** Jacobi metric, Hamilton manifold, Gutzwiller manifold, hyperbolic geometric flow, geodesic deviation, dynamical curvature, anholonomic frames, Vlasov closure, Hamiltonian stability, geometric mechanics

## Abstract

We develop a local, patchwise geometric framework that embeds a broad class of potential Hamiltonian dynamical systems into a family of Riemannian *Hamilton patches* built over an underlying Gutzwiller manifold. We adopt a conformal (Jacobi) ansatz and a frame-adapted reconstruction procedure, through which we construct, on each patch, a pulled-back metric, along with a reduced (truncated) connection (not a metric-compatible connection) and a corresponding dynamical curvature tensor governing geodesic deviation in the Hamilton coordinates. Then, using the Poisson–Hodge reconstruction, we reconstruct coordinate potentials, enforcing harmonic obstructions, and along with exactness and Jacobian nondegeneracy conditions, we obtain explicit elliptic bounds that control the connection and curvature residuals. On the basis of this construction, we formalize the notion of a *Hamilton manifold* such that reparametrized geodesics approximate Newton trajectories with controlled acceleration and tolerances. As a generalized structural framework, to promote the local Jacobi reconstructions to a coherent dynamical evolution and provide a dynamical closure, we introduce a patchwise hyperbolic geometric flow for the pullback metric coupled to a kinetic (Vlasov) closure that controls reconstruction and curvature residuals. Under natural regularity, ellipticity, and overlap-tolerance assumptions, together with precise estimates that control the reconstruction and curvature errors, we establish short-time well-posedness of the coupled Vlasov–hyperbolic geometric flow that defines the patchwise Hamilton manifold. Motivated by this construction of the *Hamilton manifold with atlas-dependent time*, we propose convergence and stability conjectures for dissipative and conservative (non-dissipative) hyperbolic geometric flows. On a single patch, these conjectures characterize local orbital stability (in the sense of coercivity modulo symmetry) and identify local linear instability when unstable linear modes are present. On a finite atlas (the *Hamilton manifold with atlas-dependent time*), we state conjectures under which local stability propagates to global stability, provided that overlap residuals remain uniformly sufficiently small. The framework identifies the geometric origin of local instability diagnostics used in Hamiltonian mechanics and outlines a practical strategy for verifying stability or instability, numerically or analytically, on finite coverings of configuration space (the *Hamilton manifold*).

## 1. Introduction

We briefly review the work of Horwitz et al. [1], which seeks to characterize chaotic Hamiltonian systems by analyzing the curvature associated with a Riemannian metric tensor, defined via a conformal transformation.

Considering the following classical Hamiltonian, defined in a curved configuration space [1]

(1) $$H_{G}=\frac{1}{2m}g_{ij}\left(x\right)p^{i}p^{j}$$

Applying the Hamilton equations to this system leads to the corresponding geodesic equations on a Riemannian manifold

(2) $${\ddot{x}}_{l}=-\Gamma_{l}^{mn}{\dot{x}}_{m}{\dot{x}}_{n}$$

where $\Gamma_{l}^{mn}$ is the connection form. The theory of geodesics on a Riemannian manifold governed by the geometric Hamiltonian $H_{G}$ is a particular case of Hamiltonian mechanics.

The stability of the geodesic flow is locally determined by considering the separation of two nearby trajectories $\xi_{l}=x_{l}^{\prime }-x_{l}$ which correspond to the components of the geodesic deviation vector at time *t*.

We introduce a parameter γ labeling a one-parameter family of geodesics in the vicinity of a reference trajectory $x_{l}\left(t\right)$ defined by Equation (2). The deviation vector is then given by $\xi_{l}\left(t\right)=\frac{\partial x_{l}\left(\gamma ,t\right)}{\partial \gamma }{|}_{\gamma =0}$.

The second covariant derivative with respect to time, $\frac{D^{2}}{D^{2}t}$, of the deviation vector yields the geodesic deviation equation [1]

(3) $$\frac{D^{2}\xi_{i}}{Dt^{2}}=R_{i}^{jlk}{\dot{x}}_{j}{\dot{x}}_{k}\xi_{l}$$

where $R_{i}^{jlk}$ are the components of the Riemann curvature tensor and ${\dot{x}}_{j}$ denotes the velocity along the reference geodesic. The local stability of the geodesic flow is governed by the geodesic deviation equation, Equation (3), and is thus determined by the curvature of the underlying manifold, which we refer to as the *Gutzwiller* manifold [1].

Next, to develop a method for analyzing local instabilities along trajectories of general Hamiltonian systems of the potential form

(4) $$H=\delta_{ij}\frac{p^{i}p^{j}}{2m}+V\left(y\right)$$

Horwitz at al. [1] construct a geometric embedding of the dynamics into a Riemannian manifold. This is achieved by mapping the Hamiltonian flow of form with Equation (4) onto a geodesic flow through a Hamiltonian $H_{G}$ of the form in Equation (1), using a conformal transformation of Equation (5)

(5) $$g_{ij}\left(x\right)=\Phi \left(x\right)\delta_{ij},\;\Phi \left(x\right)=\frac{E}{E-V(y)}\equiv F\left(y\right)$$

The resulting conformal factor defines an effective metric, enabling the application of the geodesic deviation equation to characterize local stability. The transformation relates the coordinate sets $\left\{y^{j}\right\}$ and $\left\{x_{i}\right\}$ via the conformal factor, enabling the representation of the Hamiltonian flow as geodesic motion on a curved manifold.

Two key assumptions underlie this mapping: (1) the total energy *E* is conserved and identical for both *H* and $H_{G}$, and (2) the momentum $p^{i}$ is identical in both dynamical systems governed by the respective Hamiltonians $H_{G}$ and *H*, corresponding to curved and flat spaces, respectively. This yields the relation

(6) $$E-V\left(y\right)=\delta_{ij}\frac{p^{i}p^{j}}{2m}$$

connecting the potential and kinetic terms across the two formulations.

Applying Hamilton’s equations to both $H_{G}$ and *H*, we obtain

(7) $$\begin{array}{c} {\dot{x}}_{i}=\frac{\partial H_{G}}{\partial p^{i}}=\frac{1}{m}g_{ij}p^{j}\\ {\dot{y}}^{i}=\frac{1}{m}p^{i}=\frac{\partial H}{\partial p^{i}} \end{array}$$

and by equating momenta, we find

(8) $${\dot{y}}^{j}=g^{ji}{\dot{x}}_{i}$$

which defines a local tangent space transformation. This mapping reflects a local geometric embedding of the original Hamiltonian motion. However, since the transformation is not globally integrable, it does not establish a global coordinate relation between $\left\{y^{j}\right\}$ and $\left\{x_{i}\right\}$ [1]. Using the local relation in Equation (8), we compute the second time derivative and obtain

(9) $${\ddot{x}}_{l}=g_{lj}{\ddot{y}}^{j}+\frac{\partial g_{lj}}{\partial x_{n}}{\dot{x}}_{n}{\dot{y}}^{j}$$

Substituting Equation (9) into the geodesic equation (Equation (2)), one obtains an evolution equation for $y^{l}$ in the form

(10) $${\ddot{y}}^{l}=-M_{mn}^{l}{\dot{y}}^{m}{\dot{y}}^{n}$$

with an effective connection defined by

(11) $$M_{mn}^{l}=\frac{1}{2}g^{lk}\frac{\partial g_{nm}}{\partial y^{k}}$$

This equation has the canonical structure of a geodesic equation on a manifold with a reduced, purely covariant connection. The evolution of $y^{l}$ thus mimics “free motion” (i.e., geodesic flow) under this induced connection. The geodesic-like second-order equation (Equation (10)) arises naturally in the $\left\{y^{j}\right\}$ coordinates, and we therefore identify this coordinate space as the *Hamilton manifold*. The effective connection $M_{mn}^{l}$, derived from the conformal structure induced by the transformation from $\left\{x_{i}\right\}$, governs the local dynamics on this manifold. Thus it is the Hamilton manifold that inherits the effective geometric (geodesic) embedding. In contrast, the $\left\{x_{i}\right\}$ coordinates, which facilitate the conformal embedding, define the *Gutzwiller manifold*, serving as the geometric background from which the Hamiltonian geodesic structure emerges and where the geometric properties, encoded in the connection and curvature, govern the local stability of trajectories.

Following the work of Horwitz et al. [2], and as argued in [1] using power series expansion methods, the two coordinate systems $\left\{x_{i}\right\}$ and $\left\{y^{j}\right\}$ represent two distinct coordinatizations of the dynamics. The coordinates $\left\{y^{j}\right\}$ define the Hamilton manifold, while $\left\{x_{i}\right\}$ define the Gutzwiller manifold, each associated with distinct connection forms and related locally via $\delta y^{j}:=g^{ji}\delta x_{i}$. A curvature associated with the Hamilton manifold can be derived by considering the covariant derivative of a covariant tensor defined on the Gutzwiller manifold, as constructed by Horwitz et al. [1]. Starting from the connection form on the Gutzwiller manifold, one defines a covariant derivative in the Hamilton manifold of the form

(12) $$A^{m;q}=\frac{\partial A^{m}}{\partial x_{q}}-\Gamma_{k}^{mq}A^{k}$$

where $\Gamma_{k}^{mq}$ is the connection associated with the Gutzwiller manifold. An induced connection form is obtained by lowering the index *q* using the metric $g_{lq}$, giving

(13) $$\Gamma_{lk}^{m}\equiv g_{lq}\Gamma_{k}^{mq}=\frac{1}{2}g^{mq}\left(\frac{\partial g_{lq}}{\partial y^{k}}-\frac{\partial g_{kq}}{\partial y^{l}}-\frac{\partial g_{kl}}{\partial y^{q}}\right)$$

This induced connection form is antisymmetric in its lower indices (*l*,*k*), indicating the presence of torsion. However, when evaluated along geodesic motion, the antisymmetric contributions cancel, yielding the symmetric connection form introduced in Equation (10) [1].

These derived expressions for the geodesic equation and the associated connection on the Hamilton manifold are not metric-compatible in the usual Riemannian sense. Rather, they arise from performing parallel transport in the local flat tangent space of the Gutzwiller manifold, whose metric is $g_{ij}$. Under a conformal transformation, the raising of the tensor index produces the reduced, or “truncated”, connection $M_{mn}^{l}$ given in Equation (10) [1].

To analyze the rate of transport of geodesic deviation in the Hamilton manifold, one constructs a covariant derivative consistent with the connection $M_{mn}^{l}$, which respects the induced geometric structure. The resulting second-order geodesic deviation equation is

(14) $$\frac{D^{2}\xi^{l}}{Dt^{2}}=R_{qmn}^{l}{\dot{y}}^{q}{\dot{y}}^{n}\xi^{m}$$

where the tensor $R_{qmn}^{l}$, termed the dynamical curvature [1], is given by

(15) $$R_{qmn}^{l}=\frac{\partial M_{qm}^{l}}{\partial y^{n}}-\frac{\partial M_{qn}^{l}}{\partial y^{m}}+M_{qm}^{k}M_{nk}^{l}-M_{qn}^{k}M_{mk}^{l}$$

This dynamical curvature governs the local stability of trajectories in the Hamilton manifold and generalizes the standard Riemann curvature tensor to the conformally induced structure arising from the underlying Hamiltonian dynamics.

Horwitz et al. [1,3] conjectured that the structure of Equation (14) can serve as a local criterion for diagnosing the stability of the original Hamiltonian dynamics. Specifically, the dynamical curvature tensor $R_{qmn}^{l}$ identifies local regions of instability, which are proposed to play a key role in the onset of chaotic behavior. In the classical regime, instability is indicated when at least one eigenvalue of the dynamical curvature is negative.

This paper develops a patchwise geometric framework that embeds potential Hamiltonian dynamics into locally reconstructed Jacobi metrics and then promotes those reconstructions to a coherent dynamical theory by coupling a hyperbolic geometric flow to a kinetic (Vlasov) closure. Our purpose is to (1) give a constructive procedure for producing Hamilton patches with explicit error bounds, (2) show how those patches fit into an atlas whose overlaps are controlled, and (3) introduce short-time well-posedness for the metric evolution. Then, we formulate stability and convergence criteria that connect local curvature diagnostics to global behavior.

In view of the physical interpretation, our aim is to construct a Hamilton manifold as a dynamical configuration-space geometry, where each patch’s pulled (Jacobi) metric defines an effective distance between configurations, i.e., regions of strong curvature concentrate or disperse trajectories and thereby determine which families of paths dominate the dynamics. The hyperbolic flow makes the geometry active, linking observable dynamical signatures, i.e., transition rates (the frequency at which the system moves from one region of configuration space to another), response to perturbations (how the system’s state or observables change after a small, controlled disturbance, i.e., how strongly and how fast the system reacts to small disturbances), and coherence times (the time scale over which a family of nearby trajectories remains similar or correlated before they spread apart significantly) to concrete geometric diagnostics and to a self-consistent evolution law in which geometry both encodes and reacts to the ensemble dynamics.

The remainder of this paper is structured as follows. In Section 2, the geometric foundations and local objects are addressed, introducing the Jacobi (Gutzwiller) embedding and an anholonomic frame. Then, we define the pulled (Hamilton) metric *G*, frame momenta, truncated (non-metric) connection, and the dynamical curvature. In Section 3, we introduce the patch reconstruction (Horwitz–Yahalom and Poisson–Hodge), converting sampled geodesic data into local coordinate potentials via the Horwitz–Yahalom recursion and Poisson–Hodge elliptic reconstruction. We state the acceptance conditions for a Hamilton patch (Jacobian nondegeneracy, exactness residuals, conformal compatibility) and derive explicit residual bounds that control metric and curvature errors. In Section 4, we assemble these patches into the Hamilton manifold and formulate the patchwise hyperbolic evolution for the pulled metrics (DeTurck gauge) coupled to a kinetic closure that supplies the forcing from phase-space moments. Then, we state the main conditional short time well-posedness theorem under precise regularity and overlap hypotheses, outlining the proof strategy (gauge fixing, energy estimates, Lipschitz forcing bounds, and Picard iteration). In Section 5, stability, propagation, and conjectures are introduced. We attempt to formulate conjectures and criteria linking local curvature diagnostics (dynamical curvature eigenvalues) to orbital stability or instability on a single patch and explain conditions under which local stability propagates across a finite atlas when overlap residuals are uniformly small.

## 2. Curvature in Hamiltonian Mechanics and the Hyperbolic Geometric Flow

This work develops a rigorous, patchwise geometric framework that attempts to link classical Hamiltonian dynamics to reconstructed Jacobi metrics on configuration space. As we shall show throughout this work, we construct local Hamilton patches, starting with introducing a pointwise anholonomic frame to highlight the effect of frame anholonomy on the symplectic and Hamiltonian structures, and derive quantitative estimates for the residuals that control metric, connection, and transition compatibility. We attempt to (1) provide a procedure for reconstructing a Jacobi metric from local dynamical data with explicit error bounds and (2) analyze the effects of anholonomy, sampling, and atlas coherence on the existence and stability of the resulting *Hamilton manifold*. Throughout our work, we suggest regularity hypotheses, local and global reconstruction via controlled overlaps, and estimates.

### 2.1. Local Hamiltonian Structure over a Gutzwiller Manifold: An Anholonomic Frame Approach

The following definition formalizes the geometric setting in which the classical Hamiltonian dynamics of interest coincide with geodesic motion. By identifying the kinetic Hamiltonian $H_{G}=\frac{1}{2m}p_{i}g^{ij}\left(x\right)p_{j}$ with the energy function generating the geodesic flow on a Riemannian manifold (M,g), we obtain a phase-space picture in which variational, spectral, and semiclassical constructions can be carried out intrinsically. Geometrically, we construct a framework that makes the cotangent bundle $T^{*}M$ the natural phase space, endows it with the canonical Liouville form θ and symplectic form ω=dθ, and allows one to derive geodesics, Poisson brackets, and Hamiltonian flows directly from the metric *g*. Definition 1 below defines and states this identification and the regularity conventions used throughout the paper.

**Definition** **1.** *Gutzwiller Geometry: Let $H_{G}$ be the geometric Hamiltonian of a given classical system, in the form $H_{G}=\frac{1}{2m}p_{i}g^{ij}\left(x\right)p_{j}$, whose orbits, described by the Hamilton–Jacobi equations, coincide with the geodesics on a Riemannian space (M,g) associated with the metric $g_{ij}$.*

Let (M,g) be a smooth, connected, n-dimensional Riemannian (or pseudo-Riemannian) manifold, which we refer to as the Gutzwiller manifold, equipped with a smooth, symmetric positive-definite metric tensor $g\in \Gamma (T^{*}M⊗T^{*}M)$, that is, a nondegenerate type (0,2) metric tensor field on the configuration space, which, in a local coordinate chart $\left\{x^{i}\right\}$, gives rise to the Riemannian metric $g=g_{ij}\left(x\right)dx^{i}⊗dx^{j}$ with inverse metric $g_{ij}\left(x\right)$ defined by $g^{ik}g_{kj}=\delta_{j}^{i}$.

The phase space of the classical mechanical system with configuration space M is the cotangent bundle $T^{*}M$, a 2n-dimensional smooth symplectic manifold, with canonical projection $\pi :T^{*}M\to M$. A point in $T^{*}M$ is denoted (x,p), where x∈M and $p\in T_{x}^{*}M$ are the fiber coordinates associated with the basis $dx^{i}$ of $T_{x}^{*}M$.

The coordinate system $\left(x^{i},p_{i}\right)$ on $T^{*}M$ is a canonical coordinate system, so that for the canonical chart, $\left(x^{i},p_{j}\right):T^{*}M\to R^{2n}$, where the canonical one-form and symplectic two-form take the standard forms: $\theta =\sum_{i=1}^{n}p_{i}dx^{i}$$\left(\theta_{\left(x,p\right)}\left(V\right)=p\left(d\pi \left(V\right)\right),V\in T_{\left(x,p\right)}\left(T^{*}M\right)\right)$, $\omega =d\theta =\sum_{i=1}^{n}dx^{i}\wedge dp_{i}$, closed (dω=0), nondegenerate two-form (the symplectic form; for every x∈M, the map $v\to i_{v}\omega$ is injective on $T_{x}M$).

Assume that $H_{G}:T^{*}M\to R$ is a smooth function and let the geodesic flow be driven by $H_{G}$, generated by the Hamiltonian vector field $X_{H}$ defined by $i_{X_{H_{G}}}\omega =dH_{G}$. The flow of $X_{H_{G}}$ preserves ω, meaning $L_{X_{H_{G}}}\omega =0$. This structure induces a Poisson bracket on the algebra of smooth functions $C^{\infty }\left(T^{*}M\right)$, making it a Poisson algebra. The Poisson bracket structure on $T^{*}M$ is defined by $\left\{f,g\right\}=\omega \left(X_{f},X_{g}\right)$, and in local canonical coordinates, by the property $\left\{x^{i},p_{j}\right\}=\delta_{j}^{i},\left\{x^{i},x^{j}\right\}=0,\left\{p_{i},p_{j}\right\}=0$.

Next, the x-coordinates (Gutzwiller chart) are useful as a background, since the Horwitz tangent space map (Equation (8)) is, in general, only a local and globally nonintegrable relation, so working solely in the Gutzwiller x-coordinates conceals the geometric obstructions that arise from frame anholonomy. Therefore, the following definition adopts an anholonomic coframe to record, pointwise, the structure functions (torsion/anholonomy) that alter the algebraic form of the symplectic two-form and the Hamilton equations. Keeping these terms explicit is essential to control how anholonomy affects the reconstructed Poisson structure and dynamics.

We adopt a general algebraic construction with canonical pairing, defined by a pointwise matrix inversion S·E=I, which produces anholonomic coframes with nonzero structure functions. Then, we derive the induced Poisson algebra of frame momenta, along with the anholonomy coefficients, the frame form of Hamilton’s equations, and the Levi–Civita connection in frame indices (metric plus C-terms). This construction marks the anholonomic Poisson algebra on $T^{*}U$, valid on every chart U⊂M as a local coverage.

We introduce below a pointwise frame formulation, attempting to make the Hamiltonian and local structure of the Gutzwiller manifold explicit while allowing freedom in topology and coordinate selection. Working fiberwise with a smooth coframe $e_{i}^{a}\left(x\right)$ and the induced frame momenta $p_{a}^{\prime }$, we seek to single out the geometric effects of anholonomy (structure functions $C_{bc}^{a}$) and make the phase-space description compatible with label-space constructions used in the reconstruction of Jacobi metrics. The local framework for stability and residual estimates are shown below.

**Definition** **2.** Anholonomic Gutzwiller Manifold: A Pointwise Frame Approach on Local Charts: Let (M,g) be a smooth, connected, n-dimensional Riemannian (or pseudo-Riemannian) manifold, referred to as the Gutzwiller manifold, equipped with a smooth, symmetric, nondegenerate metric tensor field $g\in \Gamma (T^{*}M⊗T^{*}M)$ as before.

Fix local coordinates $x^{k}$ on an open set (patch) U⊂M and let $g_{ij}\left(x\right)$ denote the metric components with inverse $g^{ij}\left(x\right)$. Coordinates on the cotangent bundle $T^{*}M$ are $\left(x^{i},p_{i}\right)$ with canonical Poisson brackets as before

(16) $$\left\{x^{i},p_{j}\right\}=\delta_{j}^{i}\;,\left\{x^{i},x^{j}\right\}=0\;,\left\{p_{i},p_{j}\right\}=0$$

*Let ${\left\{Y_{i}\right\}}_{i=1}^{n}$ be a local frame of vector fields on an open set U⊂M, and ${\left\{\eta^{i}\right\}}_{i=1}^{n}$ its dual coframe, satisfying*

(17) $$\eta^{i}\left(Y_{j}\right)=\delta_{j}^{i}\;,\left[Y_{i},Y_{j}\right]=C_{ij}^{k}Y_{k}$$

*where $C_{ij}^{K}$ are the structure functions on U, encoding the anholonomy of the frame.*

*Let S(x) be a smooth pointwise invertible n×n matrix field on a chart U⊂M (a coordinate patch) S:U→GL(n,R) ($S\left(x\right)\in C^{\infty }\left(U,GL\left(n\right)\right)$) and set $E:=S^{-1}$ to denote its pointwise inverse. In components*

(18) $$S_{i}^{a}\left(x\right)E_{b}^{i}\left(x\right)=\delta_{b}^{a}\;,E_{a}^{i}\left(x\right)S_{j}^{a}\left(x\right)=\delta_{j}^{i}$$

*i.e, the algebraic canonical pairing, $S\left(x\right)E\left(x\right)=I_{n}$ for every x∈U, enforced pointwise.*

*Define the frame and coframe by*

(19) $$\eta^{a}\left(x\right)=S_{i}^{a}\left(x\right)dx^{i}\;,Y_{a}\left(x\right)=E_{a}^{i}\left(x\right)\partial_{x^{i}}\;,\eta^{a}\left(Y_{b}\right)=\delta_{b}^{a}$$

*for every x∈U. For each fixed x∈U, define the linear isomorphism of label spaces*

(20) $$\begin{array}{c} S\left(x\right):R_{x}^{n}\to R_{y}^{n}\;,S\left(x\right):e_{i}\to S_{i}^{a}\left(x\right)e_{a}\\ E\left(x\right)=S^{-1}\left(x\right):R_{y}^{n}\to R_{x}^{n} \end{array}$$

*where $e_{i}$ and $e_{a}$ denote standard bases (basis vectors) of the coordinate and abstract label spaces, respectively. This is a pointwise (fiberwise) identification; the base label coordinates $y^{a}\left(x\right)$ are the abstract coordinates (label components) on $R_{y}^{n}$ (new coordinate labels on the label space $R_{y}^{n}$) related to the coordinate label space by the pointwise linear map S(x) (a pointwise linear identification encoded by S(x) and E(x)).*

*Define the matrix field S(x) by the pointwise inverse of the metric matrix:*

(21) $$S\left(x\right):=g\left(x\right)\;,S_{i}^{a}\left(x\right):=g_{ai}\left(x\right)$$

*where $g_{ij}\left(x\right)$ is the coordinate representation of the metric and $g^{ij}\left(x\right)$ is its inverse. Then its inverse has components*

(22) $$\begin{array}{c} E_{a}^{i}\left(x\right)=g^{ia}\left(x\right) \end{array}$$

*Using these matrices to define the coframe $\eta^{a}$ and the dual frame $Y_{a}$ on U by*

(23) $$\eta^{a}\left(x\right):=g_{ai}\left(x\right)dx^{i},\;Y_{a}\left(x\right):=g^{ia}\left(x\right)\partial_{x^{i}}$$

*The coframe and frame are dual pointwise:*

(24) $$\eta^{a}\left(Y_{b}\right)=S_{i}^{a}E_{b}^{i}=g^{ai}g_{ib}=\delta_{b}^{a}\;,\forall x\in U$$

Define on $T_{x}^{*}U$ frame-adapted (quasi-) momenta pointwise (fiber-linear coordinate functions) by

(25) $$p_{i}^{\prime }\left(x,p\right):=E_{i}^{a}\left(x\right)p_{a}=g^{ai}\left(x\right)p_{a}$$

*Define the bundle isomorphism (pointwise in the base) as fiberwise linear*

(26) $$\Psi_{x}:T_{x}^{*}U\to U\times R_{y}^{n}\times R_{p^{\prime }}^{n}$$

*a smooth family ${\left\{\Psi_{x}\right\}}_{x\in U}$ of linear maps depending on x.*

*If $\eta^{a}$ is exact, then the $y^{a}$ are genuine local coordinates on U and under the algebraic pairing axiom $S\left(x\right)E\left(x\right)=I_{n}$,*

(27) $$\left\{y^{a},y^{b}\right\}=0,\;\left\{y^{a},p_{b}^{\prime }\right\}=\delta_{b}^{a}$$

*where the mixed bracket is the transported canonical pairing and holds only where local primitives $y^{a}$ exist.*

*The bracket of frame momenta is*

(28) $$\left\{p_{a}^{\prime },p_{b}^{\prime }\right\}=C_{ab}^{c}\left(x\right)p_{c}\;,C_{ab}^{c}\left(x\right):=S_{j}^{c}\left(E_{a}^{i}\partial_{i}E_{b}^{j}-E_{b}^{i}\partial_{i}E_{a}^{j}\right)$$

*These variables are not globally canonical unless the frame is holonomic.*

Define the canonical Liouville one-form on $T^{*}U$ by $\theta :=p_{i}dx^{i}$. The Liouville one-form equality (pointwise identity) holds algebraically (algebraic representation) for every x;

(29) $$\theta =p_{i}dx^{i}=p_{a}^{\prime }\eta^{a}$$

*Thus, *Ψ *preserves the Liouville form pointwise on each fiber (it is a fiberwise linear change in variables that preserves the cotangent pairing).*

*Taking dθ yields the canonical symplectic form in frame coordinates (variables):*

(30) $$\omega =d\theta =dp_{a}^{\prime }\wedge \eta^{a}+p_{a}^{\prime }d\eta^{a}=dp_{a}^{\prime }\wedge \eta^{a}-\frac{1}{2}p_{a}^{\prime }C_{bc}^{a}\eta^{b}\wedge \eta^{c}$$

*Thus, although $\theta =p_{a}^{\prime }\eta^{a}$ holds algebraically for S=g, anholonomy appears in ω.*

*Introduce the frame metric and its inverse*

(31) $$G^{ab}\left(x\right):=S_{i}^{a}\left(x\right)S_{j}^{b}\left(x\right)g^{ij}\left(x\right)\;,G_{ab}\left(x\right)={\left(G^{ab}\right)}^{-1}$$

*Define the Hamiltonian*

(32) $$H_{G}^{\prime }:=\frac{1}{2m}G^{ab}\left(x\right)p_{a}p_{b}$$

The Hamiltonian vector field $X_{H_{G}^{\prime }}\in X\left(T^{*}U\right)$ is defined by

(33) $$i_{X_{H_{G}^{\prime }}}\omega :=dH_{G}^{\prime }$$

Local Hamiltonian flow map: there exists an open set $V\subset T^{*}U$ and ϵ>0 such that the flow map

(34) $$\Phi :\left(-\epsilon ,\epsilon \right)\times V\to T^{*}U\;,\Phi \left(t,z\right)=\phi_{t}\left(z\right)$$

*is smooth and satisfies $\partial_{t}\Phi \left(t,z\right)=X_{H_{G}^{\prime }}\left(\Phi \left(t,z\right)\right)$ and Φ(0,z)=z.*

*Projecting the flow to the base yields the local geodesic map*

(35) Γ:(−ϵ,ϵ)×V→U,Γ(t,z)=π∘Φ(t,z)

*and the projected curves $\gamma_{z}\left(t\right)=\Gamma \left(t,z\right)$ satisfy the geodesic equation for γ when momenta are eliminated.*

*Equivalently, expressed in the reconstructed label coordinates $y^{a}$ (when they exist), the second-order equation takes the form*

(36) $${\ddot{y}}^{a}+{\tilde{\Gamma }}_{bc}^{a}\left(y\right){\dot{y}}^{b}{\dot{y}}^{c}=0$$

*where ${\tilde{\Gamma }}_{bc}^{a}$ are the connection coefficients of the pulled metric $G_{ab}\left(x\left(y\right)\right)$ and include contributions equivalent to the anholonomy terms above.*

*All objects $G^{ab},C_{bc}^{a},{\tilde{\Gamma }}_{bc}^{a},X_{H_{G}^{\prime }},\Phi ,\Gamma$ are defined locally on the chart U. Under the regularity assumptions $g\in C^{k+2}\left(U\right)$ and $e_{i}^{a}\in C^{k+1}\left(U\right)$ (with k≥0), these objects depend smoothly on the base point x and on the metric (frame and their derivatives up to the orders indicated).*

All formulas in Definition 2 are local. Note that when the coframe is exact on a simply connected patch, one may introduce label coordinates $y^{a}$ with $dy^{a}=\eta^{a}$ and recover the usual Darboux brackets $\left\{y^{a},p_{b}^{\prime }\right\}=\delta_{b}^{a}$. Otherwise, the local fiber coordinate *y* is a pointwise label and Poisson/Hamiltonian statements must be interpreted intrinsically via ω=dθ. As anholonomy appears only through $d\eta^{a}$ (or $C_{bc}^{a}$), it modifies the algebraic form of Hamilton’s equations, but we suggest that it does not obstruct local existence or uniqueness of the Hamiltonian vector field or of local geodesics. These local estimates and regularity assumptions, as we suggest, provide the basis for the patchwise reconstruction and atlas constructions we develop subsequently.

### 2.2. Hamilton Patch and Hamilton Manifold: Patchwise Atlas and Compatibility Conditions

We introduce the Hamilton patch as the elementary, admissible local unit from which the global Hamilton manifold constructed below is assembled. Admissible patches therefore provide the controlled pieces that can be compared and assembled to produce the physically meaningful Hamilton manifold, whose metric, connection, and dynamical curvature encode effective distances and accelerations. Physically, this object may be understood as the effective configuration-space geometry that encodes how the system’s energy landscape shapes motion and stability.

In the following, we attempt to construct a local *Hamilton patch* in which the mechanical (Newtonian) dynamics and the geometric (Gutzwiller) reconstruction are made compatible to a controlled tolerance. Starting from a family of geodesics and the Horwitz–Yahalom recursion [2], we sample curvewise primitives and solve Poisson–Hodge boundary problems to produce local Hamilton coordinates $y^{a}=\phi^{a}\left(x\right)$ and a pulled metric $G_{ab}\left(y\right)$. The definition below states the quantitative regularity, nondegeneracy, and overlap tolerances (bounds) that seek to establish that (i) the reconstructed coframe is close to exact, (ii) the pulled-back metric is conformally compatible with the Jacobi metric on the patch, and (iii) curvature and connection residuals remain uniformly small so that reparametrized geodesics approximate Newtonian trajectories with controlled acceleration and errors. These patchwise statements, as suggested below, are chosen to be stable under refinement and to provide the analytic foundation underlying our approach for the subsequent kinetic averaging and stability analysis.

Note that we follow the work of Horwitz et al. [2], who develop a constructive Hamiltonian reconstruction procedure based on the Jacobi–Maupertuis correspondence and a recursion for curvewise primitives. More explicitly, given a family of real-analytic geodesics of the Jacobi (Gutzwiller) metric, their method produces convergent Taylor expansions along each geodesic and explicit primitives whose sampled values determine local coordinate potentials.

Horwitz–Yahalom provide a local, dynamical procedure that constructs coordinate labels from geodesic data. Their curvewise recursion produces a reconstructed coordinate map $x\to y^{a}\left(x\right)$ (a family of local primitives), while the symbol *y* that appears as the argument of the potential V(y) is the label space in which the mechanical potential is defined (Euclidean labels). The two become compatible only after one identifies the reconstructed labels with the potential labels via the Poisson–Hodge reconstruction and the Jacobian nondegeneracy condition. More explicitly, the Poisson–Hodge problem yields a local diffeomorphism $\Phi_{rec}:x\to y$ with nonvanishing Jacobian on the patch along with exactness residuals and orthonormal (Jacobian) misalignment residuals that are small so that the reconstructed coframe is (up to tolerance) $dy^{a}$. Under these conditions, one can identify the reconstructed labels $y^{a}\left(x\right)$ with the Euclidean labels used in V(y), and then the pulled metric and the Jacobi metric are directly comparable pointwise. Otherwise, they are distinct in a sense that one is produced from dynamics, and the other is the independent variable of the mechanical model.

Motivated by the preceding discussion, we now introduce the following construction.

**Definition** **3.** *Hamilton Patch: Horwitz–Yahalom–Hodge–Poisson Realization: Let (M,g) be an n-dimensional smooth manifold as before and let U⊂M be a coordinate chart with coordinates x. Let x(t) be a geodesic of the Gutzwiller metric. Assume dynamical equivalence (momenta equal) along the curve so the Horwitz–Yahalom recursion applies [2].*

*Assume the conformal ansatz $g_{ij}\left(x\right)=\Phi \left(x\right)\delta_{ij}$ on a coordinate chart (U,x). Let V(y) be a smooth potential on a label domain in $R_{y}^{n}$ and an energy E. Let D⊂U be a bounded, simply connected domain and $U^{\prime }\subset D$ an open subset.*

*We call $\left(U^{\prime },\phi \right)$ a* ***Hamilton patch*** *if the following hold:*

***(A) Horwitz–Yahalom curvewise data existence.*** *There exists a finite family of geodesics ${\left\{x^{\left(r\right)}\left(t\right)\right\}}_{r=1}^{m}$ through a base point in $U^{\prime }$ and ϵ>0, such that*

*1. *Φ *and V are real-analytic (or $C^{k+1}$ with k≥3) in the neighborhood of each geodesic curve, and the Horwitz–Yahalom recursion yields convergent Taylor expansions of $\Phi (x^{\left(r\right)}\left(t\right))$ for |t|<ϵ.*

*2. Curvewise primitives. For each geodesic, the curvewise primitive*

(37) $$y^{(r),a}\left(t\right)=y^{(r),a}\left(0\right)+\int_{0}^{t}\Phi^{-1}\left(x^{\left(r\right)}\left(s\right)\right){\dot{x}}^{(r),a}\left(s\right)ds$$

*are computed and sampled on $\partial U^{\prime }$ to produce Dirichlet boundary data $g^{a}$ and a set of sampled values of *Φ *on $\partial U^{\prime }$. (Normalization of primitives: Dirichlet boundary data fixes additive constants of primitives).*

***(B) Hodge/Poisson solvability and quantitative acceptance for patch reconstruction.*** *Using the interpolated $\hat{\Phi }$ (from the Horwitz–Yahalom samples) of the sampled *Φ *and the boundary data $g^{a}$, the Poisson (componentwise elliptic boundary) problems for the reconstructed coordinate potentials $\phi^{a}$ on $U^{\prime }$*

(38) $$△\phi^{a}=-▽\cdot \left({\hat{\Phi }}^{-1}dx^{a}\right)\;\mathrm{on}\;U^{\prime }\;,\phi^{a}{|}_{\partial U^{\prime }}=g^{a}$$

*admit smooth solutions $\phi^{a}\in C^{k+1}\left(U^{\prime }\right)$, satisfying the following quantitative bounds for prescribed tolerances $\delta_{J}>0,\epsilon_{r}>0,\epsilon_{\Phi }>0$:*

*1. Jacobian nondegeneracy (coordinate regularity). The reconstructed map $\phi =(\phi^{a})$ is a local diffeomorphism on $U^{\prime }$:*

(39) $$\underset{x\in U^{\prime }}{\inf}\left|det\left(\partial_{i}\phi^{a}\left(x\right)\right)\right|\geq \delta_{J}$$

*2. Exactness residual (coframe closeness). Define the reconstructed coframe $\eta^{a}:={\hat{\phi }}^{-1}\left(dx^{a}\right)$. Then the coframe coincides with $dy^{a}$ to within the residual determined by ($\left|\right|\cdot {\left|\right|}_{L^{\infty }\left(U^{\prime }\right)}$ is the uniform sup norm on $U^{\prime }$)*

(40) $$\max_{a}\left|\right|\eta^{a}-d\phi^{a}{\left|\right|}_{L^{\infty }\left(U^{\prime }\right)}\leq \epsilon_{r}\;,\eta^{a}:={\hat{\Phi }}^{-1}dx^{a}$$

*3. Conformal compatibility (dynamical compatibility). Let $\Phi_{J}\left(y\right):=\frac{E}{E-V(y)}$ denote the Jacobi conformal factor in the label coordinates y. The conformal compatibility residual satisfies*

(41) $$\underset{x\in U^{\prime }}{\sup}|\hat{\Phi }\left(x\right)-\Phi \left(x\right)|\leq \epsilon_{interp}\;and\;R_{\Phi }:=\underset{x\in U^{\prime }}{\sup}\left|\hat{\Phi }\left(x\right)-\frac{E}{E-V(\phi (x))}\right|\leq \epsilon_{\Phi }$$

*equivalently enforcing a small interpolation error.*

***(C) Stability under refinement.*** *There exists ρ>0 such that for every 0<r≤ρ, the same procedure applied to any subdomain $U^{\prime \prime }\subset U^{\prime }$ with diameter $diam(U^{\prime \prime })\leq r$ yields solutions satisfying the same bounds (possibly with tighter tolerances; this rules out pathological near-singular behavior that would force abandonment).*

***Consequences.*** *When (A)–(C) hold, $\phi :U^{\prime }\to \phi \left(U^{\prime }\right)\subset R^{n}$ is a local diffeomorphism and we set y=ϕ (ϕ defines holonomic coordinates on $U^{\prime }$). The functions $y^{a}$ are the Hamilton coordinates on $U^{\prime }$, and V(y) is the potential on the patch where the potential labels y are identified with the mechanical potential argument V(y) on $U^{\prime }$ (the dual frame coincides with coordinate vector fields $Y_{a}=g^{ia}\left(x\right)\partial_{x^{i}}=\partial_{y^{a}}$ on $U^{\prime }$) to within tolerance $\epsilon_{\Phi }$.*

*On a Hamilton patch $\left(U^{\prime },\phi \right)$, the geodesic acceleration $\ddot{y}$ is a well-defined local object [1,2]. A geodesic y(s) (affine parameter s) satisfies the local ODE on $\phi (U^{\prime })$*

(42) $$\frac{d^{2}y^{a}}{ds^{2}}+M_{bc}^{a}\left(y\left(s\right)\right)\frac{dy^{b}}{ds}\frac{dy^{c}}{ds}=0$$

*where*

(43) $$M_{mn}^{l}\left(y\right):=\frac{1}{2}g^{lk}\left(x\left(y\right)\right)\frac{\partial g_{nm}\left(x\left(y\right)\right)}{\partial y^{k}}$$

*Since $\phi \in C^{k+1}\left(U^{\prime }\right)$ and $\hat{\Phi }\in C^{k+1}\left(U^{\prime }\right)$ with k≥2 by assumption, the coefficients $M_{bc}^{a}$ are $C^{k-1}$ on $\phi (U^{\prime })$.*

***Short-time existence and uniqueness.*** *For any initial data $\left(y_{0},{\dot{y}}_{0}\right)$ with $y_{0}\in U^{\prime }$, there exists ϵ>0 and a unique smooth solution $\gamma :\left(-\epsilon ,\epsilon \right)\to U^{\prime }$ of the geodesic equation with $\gamma \left(0\right)=y_{0},\dot{\gamma }\left(0\right)={\dot{y}}_{0}$. The solution depends smoothly on initial data and extends uniquely to a maximal interval of existence.*

*Let the Riemann curvature tensor and Ricci tensor be $Ric{\left[M\right]}_{bd}=R{\left[M\right]}_{bad}^{a}$ smooth on $U^{\prime }$, where all derivatives are ordinary partials in the y-coordinates on $\phi (U^{\prime })$.*

*The difference between the reconstructed acceleration and the Newton acceleration is controlled by the connection residual. Define the connection residual*

(44) $$R_{ab}^{Mc}\left(y\right):=M_{ab}^{c}\left(y\right)-M_{ab}^{c}\left[G_{J}\right]\left(y\right)$$

*where $G_{J_{ij}}:=\Phi_{J}\left(y\right)\delta_{ij}$. Define the curvature residual tensor (componentwise difference)*

(45) $$R_{Rm_{abcd}}\left(y\right):=R_{abcd}\left[M\right]\left(y\right)-R_{abcd}\left[G_{J}\right]\left(y\right)$$

*measured in the uniform component norm on $\phi (U^{\prime })$*

(46) $$\left|\right|R_{Rm}{\left|\right|}_{L^{\infty }\left(\phi \left(U^{\prime }\right)\right)}:=\max_{a,b,c,d}\underset{y\in \phi (U^{\prime })}{\sup}\left|R_{bcd}^{a}\left[M\right]\left(y\right)-R_{bcd}^{a}\left[G_{J}\right]\left(y\right)\right|$$

*The curvature residual admits the estimate (constants $K_{i}$ as below)*

(47) $$\left|\right|R_{Rm}{\left|\right|}_{L^{\infty }\left(\phi \left(U^{\prime }\right)\right)}\leq K_{1}\epsilon_{\Phi }+K_{2}\epsilon_{r}+K_{3}\epsilon_{ortho}+K_{4}\epsilon_{interp}$$

*There exist constants $K_{1},K_{2},K_{3}>0$ depending only on ${\left||\Phi |\right|}_{C^{3}\left(U^{\prime }\right)}{,||V||}_{C^{3}\left(\phi \left(U^{\prime }\right)\right)}{,||\phi ||}_{C^{3}\left(U^{\prime }\right)}$, geometric bounds on $U^{\prime }$, and inverse powers of $\delta_{J}$. The constant $K_{4}$ appears because the interpolation error affects first derivatives of $\hat{\Phi }$ and thus second derivatives entering curvature. The constants scale polynomially with the indicated $C^{3}$ norms and with $\delta_{J}^{-p}$ for some p≥1.*

*Consequently, geodesic deviation and stability properties of mechanical trajectories are controlled locally by the patch tolerances.*

For clarity, we briefly explain some notions and notations used above. Orthonormal (Jacobian alignment) residual (frame vs. Euclidean identity) $\epsilon_{ortho}:=||S-{Id||}_{L^{\infty }}$, $S^{ab}\left(y\right):=\partial_{i}\phi^{a}\left(x\left(y\right)\right)\partial_{i}\phi^{b}\left(x\left(y\right)\right)$; Id is the n×n identity matrix in the Euclidean *y*-frame and $\epsilon_{interp}$ is the interpolation error appearing above.

These patch assumptions ensure local solvability and elliptic regularity for the Poisson–Hodge reconstruction and provide uniform control of metric and connection residuals on each patch. All statements are local and valid only while the trajectory remains inside the patch $\phi (U^{\prime })$.

Assuming the Hamilton patch tolerances are small (if the following patch assumptions hold: regularity, nondegenerate Jacobian, exactness/period control, $\hat{\Phi }\approx \Phi_{J}\circ \phi$, and turning-point gap), then, following our suggested framework, the reconstructed geodesic acceleration (after reparametrization) approximates the Newton acceleration up to a controlled error on the patch.

The following definition formalizes the notion of a *Hamilton manifold*. The purpose is to state when a local Hamiltonian reconstruction can be assembled consistently into a global structure on $M_{H}$ and to identify the small residuals that must be bounded to obtain a global Jacobi metric.

**Definition** **4.** *Hamilton Manifold: Let M be a smooth n-manifold as before, equipped with a conformal chartwise ansatz $g_{ij}\left(x\right)=\Phi \left(x\right)\delta_{ij}$ as before, on coordinate charts (U,x). Fix a smooth potential $V:Y\subset R^{n}\to R$ and an energy E.*

*A* ***Hamilton manifold*** *for (V,E) is a triple*

(48) $$H=(M_{H},g,A)$$

*consisting of an open domain $M_{H}\subseteq M$, the Riemannian metric g as before, and an atlas $A={\left\{\left(U_{\alpha },\phi_{\alpha }\right)\right\}}_{\alpha \in I}$ of Hamilton patches as before, covering $M_{H}$ that satisfy the conditions below.*

*For each α, the pair $\left(U_{\alpha },\phi_{\alpha }\right)$ is a Hamilton patch in the sense of the Hamilton patch definition (A)–(C) together with the explicit assumptions of regularity, Jacobian nondegeneracy, period control, conformal (Jacobi) compatibility, interpolation, and orthonormal residual.*

***Transition compatibility and atlas coherence.*** *For every ordered pair of patches, with nonempty overlap $U_{\alpha }\cap U_{\beta }\neq 0$, define the transition map*

(49) $$T_{\alpha \beta }:=\phi_{\beta }\circ \phi_{\alpha }^{-1}:\phi_{\alpha }\left(U_{\alpha }\cap U_{\beta }\right)\to \phi_{\beta }\left(U_{\alpha }\cap U_{\beta }\right)$$

*where it is a $C^{k}$ diffeomorphism (with the same k used in patch regularity) and satisfies $\left|\right|T_{\alpha \beta }{\left|\right|}_{C^{k}}<\infty$.*

*Require the following small transition residual (quantitative compatibility) on overlaps.*

***Transition regularity.*** *There exists a prescribed tolerance $\epsilon_{T,\alpha \beta }$ (derived from patch tolerances) such that $T_{\alpha \beta }$ is a $C^{k}$ diffeomorphism and on the overlap*

(50) $$\left|\right|T_{\alpha \beta }-Id{\left|\right|}_{C^{1}\left(\phi_{\alpha }\left(U_{\alpha }\cap U_{\beta }\right)\right)}\leq \epsilon_{T,\alpha \beta }$$

*This ensures coordinate alignment and that Jacobian misalignment is controlled.*

***Metric pullback compatibility.*** *The Hamilton manifold requires that on each overlap, the reconstructed metrics are mutually compatible up to controlled residuals. The family $\left\{G_{\alpha }\right\}$ defines a reconstructed Riemannian structure on the label side. The reconstructed pulled metrics $G_{\alpha }$ and $G_{\beta }$ (expressed in the same y-frame via $T_{\alpha \beta }$, pulling $G_{\beta }$ by $T_{\alpha \beta }$ to the $\phi_{\alpha }$ frame) satisfy*

(51) $$\left|\right|G_{\alpha }-T_{\alpha \beta }G_{\beta }{\left|\right|}_{C^{1}\left(\phi_{\alpha }\left(U_{\alpha }\cap U_{\beta }\right)\right)}\leq \epsilon_{G,\alpha \beta }\left(\epsilon_{\Phi ,\alpha },\epsilon_{\Phi ,\beta },\epsilon_{r},\epsilon_{ortho}\right)$$

*with $\epsilon_{G,\alpha \beta }$ controlled by the patch residuals $\epsilon_{\Phi ,\alpha },\epsilon_{\Phi ,\beta },\epsilon_{r,\alpha },\epsilon_{r,\beta },\epsilon_{ortho}$.*

***Connection and curvature compatibility.*** *The connection and curvature residuals on the overlap satisfy*

(52) $$||M\left[G_{\alpha }\right]-T_{\alpha \beta }M\left[G_{\beta }\right]{\left|\right|}_{L^{\infty }}\leq \epsilon_{M,\alpha \beta }\;,||R\left[G_{\alpha }\right]-T_{\alpha \beta }R\left[G_{\beta }\right]{\left|\right|}_{L^{\infty }}\leq \epsilon_{Rm,\alpha \beta }$$

*with the ϵ constants small and controlled by the patch tolerances.*

***Potential and Jacobi compatibility.** The family $\left\{G_{J,\alpha }\right\}$ defines the ideal Jacobi metric charts. The Jacobi factor pulled back via the two charts must agree up to tolerance*

(53) $$\left|\right|\Phi_{J}\circ \phi_{\alpha }-\left(\Phi_{J}\circ \phi_{\beta }\right)\circ T_{\alpha \beta }{\left|\right|}_{L^{\infty }\left(\phi_{\alpha }\left(U_{\alpha }\cap U_{\beta }\right)\right)}\leq \epsilon_{\Phi ,J,\alpha \beta }$$

*When the residuals are driven to zero under refinement, the reconstructed metric atlas converges to a globally defined Jacobi metric $G_{J}$ on $M_{H}$.*

*Equivalently, V evaluated at the two label maps must match on overlaps to within the tolerance used as before.*

***Dirichlet/normalization consistency.*** *Additive constants for primitives and the Dirichlet boundary data used to define $\phi_{\alpha },\phi_{\beta }$ must be consistent on overlaps: if $g_{\alpha }^{a},g_{\beta }^{a}$ are the boundary data, then on $U_{\alpha }\cap U_{\beta }$*

(54) $$\left|\right|g_{\alpha }^{a}-g_{\beta }^{a}\circ T_{\alpha \beta }{\left|\right|}_{C^{0}}\leq \epsilon_{g,\alpha \beta }$$

***Cocycle and atlas coherence (global consistency).*** *To obtain a genuine atlas, one must also enforce the cocycle condition on triple overlaps: For $U_{\alpha }\cap U_{\beta }\cap U_{\gamma }\neq ⌀$,*

(55) $$T_{\beta \gamma }\circ T_{\alpha \beta }=T_{\alpha \gamma }$$

*up to a small tolerance in $C^{1}$:*

(56) $$\left|\right|T_{\beta \gamma }\circ T_{\alpha \beta }-T_{\alpha \gamma }{\left|\right|}_{C^{1}}\leq \epsilon_{cocycle}$$

*Small cocycle errors can be driven to zero under refinement; if they persist, they indicate an obstruction to forming a smooth global atlas.*

***All overlap tolerances are controlled by the patch tolerances.*** *Under refinement they may be driven to zero so that A defines a smooth atlas on $M_{H}$. The Hamilton domain $M_{H}\subseteq M$ is the maximal open set admitting an atlas A of Hamilton patches satisfying the above conditions. If such an atlas covers all of M then (M,g,A) is a global Hamilton manifold for the potential V and energy E.*

The conditions above are defined such that they can remain stable under refinement. If one makes the patch tolerances arbitrarily small, then it forces the errors in transitions, the metric, the connection, and the Jacobi residuals to vanish, resulting in a global Jacobi metric on $M_{H}$. The cocycle and Dirichlet consistency requirements identify the obstructions to forming a smooth atlas. Persistent cocycle errors imply topological or sampling obstructions. The resulting atlas either extends to all of M (yielding a global Hamilton manifold) or else identifies the maximal domain $M_{H}$ on which a coherent Hamilton reconstruction exists.

Definition 4 describes the Hamilton manifold as the data reconstructed configuration-space geometry that summarizes how the system’s energy landscape shapes motion and stability. Its pulled (Jacobi) metric encodes energy-weighted distances that determine preferred directions and travel times (the time integral along a path). The associated connection prescribes the local accelerations experienced by trajectories, and the dynamical curvature diagnoses whether nearby paths will converge or diverge (local stability or instability). This manifold is the observable, reconstructible scaffold inferred from local measurements, i.e., a diagnostic object that yields local predictions about trajectories and sensitivity to perturbations.

### 2.3. Atlas-Dependent Time and Hyperbolic Geometric Flow

The embedding is a mapping from potential Hamiltonian dynamics to a geometric picture. To study THE time evolution of trajectories, one must integrate the original Hamiltonian equations separately. Stability analysis on the manifold is diagnostic and local. There is no causal propagation of “geometric signals”; i.e., any inference about future behavior is limited to very short times. Predictability is constrained by the reconstruction error and by the fact that the geometry does not “respond” to the ensemble.

Next, we discuss the dynamical bridge between geometric diagnostics and the time evolution of the potential Hamiltonian. The purpose is to convert static curvature indicators into causal (distinguishing local transient sensitivity from instabilities that will causally invade other patches) and finite speed processes driven by ensemble feedback, thereby enabling principled, short-time predictions about whether local sensitivity will remain local or develop into global instability (capable of capturing feedback-driven global reorganizations).

We adopt a time-dependent atlas approach, suggesting it is the natural coordinate framework in which the geometric degrees of freedom and the underlying Newtonian/Hamiltonian dynamics evolve on the same dynamical framework.

From the Newtonian correspondence viewpoint, one may think of particle acceleration as a second order in time and, after the Jacobi reparametrization, it shows up as a hyperbolic evolution of the metric pulled into the moving frame. We suggest that if the coordinate charts follow the main particle flow, geodesics of the pulled metric reparametrized appropriately track Newton trajectories with quantitatively small residuals. From the dynamics and geometry perspective, moving charts implement inertial transport (Lie dragging) and thereby separate geometric evolution from coordinate drift. Infinitesimal symmetry motions (diffeomorphisms and reparametrizations) are absorbed into the atlas evolution, while the orthogonal, physically relevant modes remain and can be analyzed.

Time-dependent charts are essential for stability analysis. They reduce interpolation and Jacobian errors on overlaps, keep the metric coefficients and their derivatives uniformly controlled in the chosen Sobolev/Hölder norms, and thereby permit uniform energy estimates on each patch. They also provide a geometric framework for introducing modulation parameters and enforce orthogonality to the finite-dimensional neutral subspace. In short, a time-dependent atlas both reflects the physical inertial coupling between particles and geometry and provides the analytic structure (reduced residuals, uniform coefficients, and natural modulation coordinates) needed to carry out rigorous short-time existence and conjectured orbital stability arguments for the hyperbolic geometric flow.

In our time-dependent patchwise formulation, we follow Rajeev’s work [4] and its main ideas; it generalizes Riemannian curvature to Hamiltonian phase space and shows how momentum-dependent curvature tensors, when averaged against a phase-space density, produce the familiar gravitational, electromagnetic, and scalar actions.

Rajeev’s construction extends the notion of curvature from configuration space to the full symplectic phase space (*q*,*p*). He defines a momentum-contracted curvature $R_{ab}\left(q,p\right)$ built from derivatives of the geometric Hamiltonian (the Gutzwiller metric), which reduces to the Riemann tensor in the Riemannian geometry $R_{ij}\left(q,p\right)=-g_{im}\left(q\right)p^{k}p^{l}R_{klj}^{m}\left(q\right)$, where two of the indices of the Riemann tensor are contracted by momentum and $p^{k}=g^{kn}p_{n}$ (in accordance with the structure of the momentum defined in Equation (25)).

He shows how a Boltzmann (or Liouville) weight integrates these phase-space curvature objects to produce effective configuration-space tensors and demonstrates that the phase-space integral of the generalized Ricci tensor reproduces action terms of the Einstein–Maxwell–dilaton type.

Therefore, we follow Rajeev’s idea of a generalization of the Riemannian geometry corresponding to more general Hamiltonians and we adopt the mechanism Rajeev analyzes: (a) compute curvature objects that depend on both position and momentum, (b) integrate them against a physically motivated distribution (Vlasov), and (c) interpret the result as an effective geometric source or forcing for the metric evolution.

**Definition** **5.** *Hamilton manifold with atlas-dependent time and hyperbolic geometric flow Let M be a smooth n-manifold and let the Hamilton manifold $H=(M_{H},g,A)$ be as before.*

*Let $V:Y\subset R^{n}\to R$ and E∈R (used only to state Jacobi compatibility on patches).*

*A time-dependent Hamilton manifold for (V,E) is a tuple*

(57) $$H\left(t\right)=(M_{H},A\left(t\right),{\left\{G_{\alpha }\left(\cdot ,t\right)\right\}}_{\alpha \in I})$$

*satisfying the following:*

***(A) Atlas, coverage, and time-dependence.***

*1. $M_{H}\subset M$ is open and covered by a (finite or countable) family of patches ${\left\{U_{\alpha }\right\}}_{\alpha \in I}$.*

*2. $A\left(t\right)={\left\{\left(U_{\alpha },\phi_{\alpha }\left(t\right)\right)\right\}}_{\alpha \in I}$ is a time-dependent atlas on $M_{H}$. For each α and each t, the chart map*

(58) $$\phi_{\alpha }\left(t\right):U_{\alpha }\to V_{\alpha }\left(t\right)\subset R^{n},\;x\to y=\left(y^{a}\right)$$

*is a $C^{k}$ diffeomorphism in the spatial variable with k≥3. The maps $\phi_{\alpha }\left(t\right)$ are $C^{1}$ in t (or smoother as required by the analytic hypotheses below).*

*3. On overlaps, $U_{\alpha }\cap U_{\beta }\neq ⌀$ define the base transition*

(59) $$T_{\alpha \beta }\left(t\right)=\phi_{\beta }\left(t\right)\circ \phi_{\alpha }^{-1}\left(t\right):\phi_{\alpha }\left(U_{\alpha }\cap U_{\beta }\right)\to \phi_{\beta }\left(U_{\alpha }\cap U_{\beta }\right)$$

*assume $C^{1}$ in t and $C^{k}$ in space.*

***(B) Pulled metric on a patch.***

*1. For each α and t, the pulled metric in y-coordinates is a Riemannian metric*

(60) $$G_{\alpha }\left(y,t\right)=G_{\alpha ,ab}\left(y,t\right)dy^{a}⊗dy^{b}$$

*with $G_{\alpha ,ab}\in C_{t}^{2}C_{y}^{k}$ and $G_{\alpha }\left(\cdot ,t\right)$ positive-definite for all t in the time interval of interest.*

*2. Denote the inverse by $G_{\alpha }^{ab}\left(y,t\right)$, the Riemann tensor by $R_{bcd}^{a}$, and the Ricci tensor by $Ric_{ab}$.*

***(C) Kinetic Hamiltonian on a patch.***

1. On the cotangent patch $T^{*}U_{\alpha }$, use non-canonical coordinates $\left(y^{a},p_{a}\right)$.

*2. The kinetic (Gutzwiller) Hamiltonian is defined by*

(61) $$H_{\alpha ,G}\left(y,p\right):=\frac{1}{2m}g_{\alpha }^{ab}\left(x\left(y\right)\right)p_{a}p_{b}$$

***(D) Hyperbolic geometric flow and forcing.***

*1. Let the metric on each patch evolve by the Lie derivative along $X_{H_{\alpha ,G}}$ (the Hamiltonian flow),*

(62) $$\partial_{t}^{2}G_{\alpha ,ab}=L_{X_{H_{\alpha ,G}}}^{2}g_{\alpha ,ab}$$

*2. Equivalently, let the metric on each patch evolve by the hyperbolic geometric flow*

(63) $$\partial_{t}^{2}G_{\alpha ,ab}\left(y\right)=-2Ric{\left[G_{\alpha }\right]}_{ab}\left(x\left(y\right)\right)+F{\left[G_{\alpha }\right]}_{ab}\left(x\left(y\right)\right)$$

*defined as follows: let $Ric_{ab}\left(x\left(y\right),p\right)$ be defined by the contraction of two of the indices of the Riemann tensor by momentum (following Rajeev’s work [4]) (note that the truncated connection $M_{bc}^{a}$ is expressed in terms of the Gutzwiller metric g(x) and the reconstruction map, not necessarily as the Levi–Civita connection of the pulled metric G(y));*

(64) $$Ric_{ij}\left(x\left(y\right),p\right):=-g_{im}\left(x\left(y\right)\right)p^{k}p^{l}R_{klj}^{m}\left(x\left(y\right)\right),\;p^{k}:=g^{km}p_{m}$$

*(note that $p^{k}$ carries the same structure of the momentum defined in Equation (25)), where*

(65) $$R_{klj}^{m}\left(x\left(y\right)\right):=R_{klj}^{m}\left(M\left(x\left(y\right)\right)\right),\;M_{mn}^{l}\left(x\left(y\right)\right):=\frac{1}{2}g^{lk}\left(x\left(y\right)\right)\frac{\partial g_{nm}\left(x\left(y\right)\right)}{\partial y^{k}}$$

*such that the following kinetic coupling holds: evolve a phase-space density $f_{\alpha }\left(x\left(y\right),p,t\right)$ under $H_{\alpha ,G}$ (Vlasov) and set*

(66) $$Ric{\left[G_{\alpha }\right]}_{ab}\left(x\left(y\right)\right):=\int Ric_{ab}\left(x\left(y\right),p\right)f_{\alpha }\left(x\left(y\right),p\right)dp$$

*where $f_{\alpha }\left(x\left(y\right),p,t\right)\geq 0$ with $f\in C^{0}\left(\left[0,T\right];L^{1}⋂H_{y}^{s}L_{p}^{1}\right)$ solving the Vlasov equation*

(67) $$\partial_{t}f+\left\{f,H_{\alpha ,G}\right\}=0\;on\;T^{*}U_{\alpha }$$

*with initial data $f_{0}$. The density with respect to the coordinate volume dydp may be considered as $f_{\alpha }:=f_{can}\left(x\left(y\right),p,t\right)\left|det\frac{\partial x}{\partial y}\left(y\right)\right|=f_{pull}\left(y,p,t\right)J\left(y\right)$, where $J\left(y\right):=|det\frac{\partial x^{i}}{\partial y^{a}}|$. This is the “true density” relative to dydp. A time-dependent function defined for t∈[0,T] that is continuous in time with values in the Banach space obtained by intersecting two function spaces on phase space: the space of integrable functions in (y,p) and the space of functions whose y-regularity is Sobolev $H^{s}$ and whose $H_{y}^{s}$-norm is integrable in p. Concretely, for every t, the function belongs to both spaces and the map $t\to f_{\alpha }$ is continuous with respect to the natural norm on the intersection.*

*The forcing tensor $F_{\alpha }$ is defined by*

(68) $$F_{\alpha ,ab}\left(x\left(y\right),p\right):=\left\{H_{\alpha ,G},\left\{H_{\alpha ,G},g_{\alpha ,ab}\right\}\right\}\left(x\left(y\right),p\right)+2Ric_{ab}\left(x\left(y\right),p\right)$$

*such that*

(69) $$F{\left[G_{\alpha }\right]}_{ab}\left(x\left(y\right)\right):=\int F_{\alpha ,ab}\left(x\left(y\right),p\right)f_{\alpha }\left(x\left(y\right),p\right)dp$$

***(E) Patch compatibility.*** *Overlaps $U_{\alpha }⋂U_{\beta }$ require controlled residuals so that the local evolutions agree up to prescribed small errors (see tolerances below).*

***(F) Relation to Newtonian dynamics (Jacobi correspondence).***

*1. Let $G_{J}\left(y\right)=\Phi_{J}\left(y\right)\delta$ denote the Jacobi metric with $\Phi_{J}\left(y\right)=\frac{E}{E-V(y)}$ on the region {V<E}. If for some patch α and time t, one has $G_{\alpha }\left(y\left(x\right),t\right)=g_{\alpha }\left(x\left(y\right)\right)=G_{J}$ exactly, then unparametrized geodesics of $G_{J}$ correspond to Newton trajectories of the potential Hamiltonian $H_{pot}=\frac{1}{2m}{\left|p\right|}^{2}+V\left(y\right)$ at energy E after reparametrization $\frac{ds}{dt}=\Phi_{J}^{-1}$.*

*2. When $G_{\alpha }\left(\cdot ,t\right)$ differs from $G_{J}$ or evolves in time, the correspondence is approximate. Quantitative residuals controlling the approximation are*

(70) $$\begin{array}{c} \delta_{\Gamma }\left(\alpha ,t\right):=||\Gamma \left[G_{\alpha }\left(\cdot ,t\right)\right]-M\left[G_{J}\right]{\left|\right|}_{C^{0}\left(V_{\alpha }\right)},\\ \delta_{L}\left(\alpha ,t\right):=\underset{\left(y,p\right)\in S_{\alpha }}{\sup}\left|\right|L_{p/m}G_{\alpha }\left(\cdot ,t\right)-\partial_{t}G_{\alpha }\left(\cdot ,t\right){\left|\right|}_{C^{0}\left(V_{\alpha }\right)} \end{array}$$

*where $S_{\alpha }$ is the chosen representative set on the energy shell. If $\delta_{\Gamma },\delta_{L}$ are small, geodesics of $G_{\alpha }\left(\cdot ,t\right)$ reparametrized by $ds/dt=\Phi_{J}^{-1}$ approximate Newton trajectories with errors controlled by $\delta_{\Gamma },\delta_{L}$ (Gronwall estimates along characteristics). Small $\delta_{L}$ means that the time dependence of G is well-aligned with advection by the particle velocity, so the geometric dynamics remain close to the potential (Newtonian) dynamics. The difference $\Gamma \left[G_{\alpha }\left(\cdot ,t\right)\right]-M\left[G_{J}\right]$ is a (1,2)-tensor field (connection coefficients themselves are not tensors, but their difference is). This tensor measures the local mismatch of affine structures: it controls the difference in geodesic accelerations produced by the two metrics. Small $\delta_{\Gamma }$ means that the two connections are close in sup norm, so geodesic equations for $G_{\alpha }$ and $G_{J}$ produce nearby accelerations.*

***(G) Analytic hypotheses and tolerances.*** Fix a time interval [0,T] and a Sobolev index s>n/2+2. There exist constants $M_{G},M_{T},M_{f},\lambda >0$ and small tolerances $\epsilon_{G},\epsilon_{F},\epsilon_{R},\epsilon_{I},\delta_{\Gamma }^{*}$, $\delta_{L}^{*}>0$, such that the following hold uniformly for all α and t∈[0,T]:

*1. Metric regularity and ellipticity:*

(71) $$\left|\right|G_{\alpha ,ab}{\left(\cdot ,t\right)||}_{H^{s}\left(V_{\alpha }\right)}+\left|\right|\partial_{t}G_{\alpha ,ab}\left(\cdot ,t\right){\left|\right|}_{H^{s-1}\left(V_{\alpha }\right)}\leq M_{G},\;G_{\alpha }\left(\cdot ,t\right)\geq \lambda Id$$

*2. Atlas regularity: for all overlaps*

(72) $$\left|\right|T_{\alpha \beta }{\left(t\right)||}_{C^{k}\left(V_{\alpha }⋂V_{\beta }\right)}+\left|\right|\partial_{t}T_{\alpha \beta }\left(t\right){\left|\right|}_{C^{k-1}}\leq M_{T}$$

*3. Metric pullback residual (overlap tolerance):*

(73) $$\epsilon_{G,\alpha \beta }:=\left|\right|G_{\alpha }-T_{\alpha \beta }^{*}G_{\beta }{\left|\right|}_{C^{1}\left(V_{\alpha }⋂V_{\beta }\right)}\leq \epsilon_{G}$$

*4. Forcing residual (overlap):*

(74) $$\epsilon_{F,\alpha \beta }:=\left|\right|F_{\alpha }-T_{\alpha \beta }^{*}F_{\beta }{\left|\right|}_{L^{\infty }\left(V_{\alpha }⋂V_{\beta }\times P\right)}\leq \epsilon_{F}$$

*where P is the chosen representative momentum set.*

*5. Curvature and inertial bounds:*

(75) $$\begin{array}{c} \epsilon_{R}\left(\alpha \right):=\underset{\left(y,p\right)\in S_{\alpha }}{\sup}||Ric\left[G_{\alpha }\right]{\left|\right|}_{H^{s-2}\left(V_{\alpha }\right)}<\infty \\ \epsilon_{F}\left(\alpha \right):=\underset{\left(y,p\right)\in S_{\alpha }}{\sup}\left|\right|F_{\alpha ,ab}\left(x\left(y\right),p\right){\left|\right|}_{H^{s-2}\left(V_{\alpha }\right)}<\infty \end{array}$$

*6. Phase-space distribution control:*

(76) $$\left|\right|f_{\alpha }{\left|\right|}_{L_{p}^{1}H_{y}^{s}}\leq M_{f},\;suppf_{\alpha }\subset \left\{H_{\alpha ,G}\leq E_{max}\right\}$$

*7. Newton compatibility tolerances:*

(77) $$\delta_{\Gamma }\left(\alpha ,t\right)\leq \delta_{\Gamma }^{*},\;\delta_{L}\left(\alpha ,t\right)\leq \delta_{L}^{*}$$

*The patchwise hyperbolic PDE for the metric is a quasilinear, second-order hyperbolic system, and under the hypotheses already stated above, it is of the same structural type of the standard short-time existence machinery (gauge fixing/DeTurck trick or reduction to symmetric hyperbolic first-order system+energy estimates) as in Kong Liu’s work [5,6].*

***(H) Short-time existence and well-posedness*** *[5,6].*

*Assume initial data $G_{\alpha }\left(\cdot ,0\right)$ and $\partial_{t}G_{\alpha }\left(\cdot ,0\right)$ are given on each patch with the regularity and ellipticity in (G) and let $\left(M^{\prime },G_{\alpha }\left(\cdot ,0\right)\right)$ be a compact Riemannian manifold. Then, for a chosen closure (kinetic coupling) and under the bounds in (G), the hyperbolic geometric flow system on each patch admits a unique short-time solution on $M^{\prime }\times \left[0,T\right]$. The solution depends continuously on the initial data and on the chosen closure parameters. The tolerances in (G) control the size of T and the stability constants in the energy estimates.*

A time-dependent atlas is a coordinate system that moves with the dynamics. Physically, this means that we choose local frames or charts that follow the dominant transport. Making the charts time-dependent turns the metric evolution into a genuinely dynamical, second-order (inertial) law on each patch, which mirrors Newtonian acceleration and the Hamiltonian transport of matter in phase space. Our construction, as proposed, may reflect the underlying physics and enhance the geometric description.

Heuristically, one may think of the manifold as a fabric that is both shaped by and carries particles. Particles push on the fabric through momentum-weighted curvature, and the fabric responds with inertial acceleration. If one rides along with the particles (use moving charts), one sees the local geometry change more slowly and coherently. The equations that govern that change are second-order in time and respect the Hamiltonian transport of matter. This is the physical rationale behind Definition 5: it makes the geometric degrees of freedom and the kinetic degrees of freedom evolve on the same dynamical framework and minimizes artificial coordinate effects (coordinate distortions) that could obscure the underlying physical behavior.

By evolving the metric using Rajeev’s general Hamiltonian curvature and phase-space moments, we have promoted the metric from a passive “snapshot” to an active medium, turning a static diagnostic into a causal, ensemble-driven field.

We made dynamical and directionally informed geometry that responds to the particle ensemble, propagates geometric disturbances at finite speed, and feeds back on particle motion.

Dynamically, this framework results in causality, i.e., disturbances propagate at finite characteristic speeds, so instability effects arrive at other patches after a “predictable delay”. Moreover, this framework supports mode structure, i.e., the system supports multiple wave families with distinct dispersion and growth properties, and results in two-way feedback. Particle moments drive geometry, the changed geometry alters geodesics and thus the moments closing a self-consistent loop that can produce amplification, saturation, or damping.

Finally, in view of the stability interpretation, the static Jacobi curvature indicates where sensitivity exists; however, in our time-dependent evolution framework, it indicates whether that sensitivity will grow, travel, or decay. Furthermore, a negative eigenvalue of curvature is a “seed”, where under the hyperbolic geometric flow, it can emit a propagating curvature front, indicating whether ensemble feedback will amplify, saturate, or quench it. Also, genuine dynamical instabilities obey the causal dispersion relations, while reconstruction noise typically will not.

The combination of dispersion analysis, causal timing, and moment correlations may be the practical approach to distinguish physics from artifact.

## 3. Local and Global Stability via Convergence-Stability Conjectures

We follow Bahuaud et al.’s work [7,8], based on the convergence-stability principle (Theorem 3.6 in [7]); if a geometric flow admits a gauging whose linearization is sectorial and the limit is strictly linearly stable, then convergence of one trajectory implies convergence of all nearby trajectories (in the weighted Hölder topology). However, it is formulated for gauged parabolic evolutions and therefore does not apply directly to a pure second order in the time hyperbolic equation. We adopt the idea behind the theorem that continuous dependence + linear stability (spectral gap) → convergence stability [9] (stability of nearby trajectories), and conjecture, that it does carry over to hyperbolic models provided one replaces the parabolic/sectorial hypotheses by the correct hyperbolic analogues (exponential decay of the linear propagator for damped waves or coercivity/modulation for conservative waves).

We adopt two admissible adaptation strategies:

1. Damped hyperbolic, including a damping term, often recovers an exponential decay property for the linearized gauged evolution (linearized damped propagator). The damped case analogue of Theorem 3.6 [7]: if the gauged damped hyperbolic flow is well-posed with continuous dependence, and the linearized gauged propagator has a uniform spectral gap (exponential decay), then small perturbations of any convergent reference trajectory produce global solutions that remain close and converge exponentially to the same limit (after undoing the gauge).
2. Conservative (non-dissipative) hyperbolic: orbital stability. The conservative analogue of Bahuaud et al. [7,8] convergence stability: continuous dependence + linear stability (in the sense given below) → orbital stability of nearby trajectories.

**Conjecture** **1.** *Local stability and instability for dissipative hyperbolic geometric flows: Let H(t) be a smooth n-manifold covered by a finite atlas $\left\{\left(V_{\alpha },\phi_{\alpha }\right)\right\}$ as before. On each patch $V_{\alpha }$, consider the patch metric $G_{\alpha }\left(y,t\right)$ and the hyperbolic geometric flow in local patch coordinates y:*

(78) $$\partial_{t}^{2}G_{\alpha ,ab}\left(y,t\right)=-2Ric{\left[G_{\alpha }\right]}_{ab}\left(x\left(y\right),t\right)+F{\left[G_{\alpha }\right]}_{ab}\left(x\left(y\right),t\right)$$

*where x=x(y) is the reconstruction map on the patch, $Ric[G_{\alpha }]$ is the Ricci tensor computed in the reconstructed x-chart as before. Fix s>n/2+2 and denote*

(79) $$X:=H^{s}\left(V_{\alpha }\right)\times H^{s-1}\left(V_{\alpha }\right)$$

*Let ${\tilde{G}}_{\alpha }\left(t\right)$ denote a reference short-time solution on a patch $I=[t_{0},t_{0}+T_{\alpha }]$ (or a global reference defined for t≥0 when stated).*

*Assume*

***(R1)** **Regularity.*** *${\tilde{G}}_{\alpha }\in C^{2}\left(\left[0,\infty \right);H^{s}\left(V_{\alpha }\right)\right)$ and*

(80) $$\underset{t\geq 0}{\sup}\left(|\right|{\tilde{G}}_{\alpha }{\left(t\right)||}_{H^{s}}+\left|\right|\partial_{t}{\tilde{G}}_{\alpha }{\left(t\right)||}_{H^{s-1}})\leq M_{0}$$

***(R2) Gauged well-posedness.*** *There exists a gauge (DeTurck or harmonic map gauge) reducing (HGF Equation (78)) to a quasilinear system for the perturbation $v_{\alpha }=G_{\alpha }-{\tilde{G}}_{\alpha }$ that is well-posed in X and depends continuously on initial data in X.*

***(R3) Dissipative linear spectral gap.*** *The linearization about ${\tilde{G}}_{\alpha }$ in first-order form generates a propagator $U_{\alpha }\left(t,s\right)$ on X and there exist constants $C_{L}\geq 1$ and μ>0, such that for all t≥s≥0*

(81) $$\left|\right|U_{\alpha }\left(t,s\right){\left|\right|}_{X\to X}\leq C_{L}e^{-\mu (t-s)}$$

***(R4) Quadratic nonlinearity.*** *The nonlinear remainder $N_{\alpha }\left(v_{\alpha }\right)$ satisfies, for $\left|\right|v_{\alpha }{\left|\right|}_{X}$ small,*

(82) $$\left|\right|N_{\alpha }\left(v_{\alpha }\right){\left|\right|}_{H^{s-2}\times H^{s-3}}\leq C_{N}\left|\right|v_{\alpha }{\left|\right|}_{X}^{2}$$

Then there exists $\epsilon_{\alpha }>0$ and constants $C,\mu^{\prime }>0$, such that for any initial perturbation $w_{\alpha }\left(0\right)\in X$ with $\left|\right|w_{\alpha }\left(0\right)\left|\right|\leq \epsilon \leq \epsilon_{*}$, the gauged dissipative patch flow has a unique global solution $w_{\alpha }\left(t\right)\in C^{0}\left(\left[0,\infty \right);X\right)$ and

(83) $$\left|\right|w_{\alpha }\left(t\right){\left|\right|}_{X}\leq Ce^{-\mu^{\prime }t}\epsilon \;\mathrm{for}\;\mathrm{all}\;t\geq 0$$

*We say ${\tilde{G}}_{\alpha }$ is locally stable on the patch $V_{\alpha }$ (or simply stable on I).*

*If, on the patch, the linearized propagator instead satisfies $\left|\right|U_{\alpha }\left(t,s\right)\left|\right|≳e^{\lambda (t-s)}$ for some λ>0, then ${\tilde{G}}_{\alpha }$ is locally unstable on that patch in the sense of the following local instability definition.*

*A reference solution ${\tilde{G}}_{\alpha }\left(t\right)$ is locally unstable on the patch $V_{\alpha }$ over the interval I if one of the following equivalent conditions holds.*

*1. There exists a sequence of initial perturbations $w_{n}\left(t_{0}\right)\in X$ with $\left|\right|w_{n}\left(t_{0}\right){\left|\right|}_{X}\to 0$, such that the corresponding solutions $w_{n}\left(t\right)$ of the linearized (or full nonlinear) gauged perturbation problem satisfy*

(84) $$\underset{t\in I}{\sup}\left|\right|w_{n}\left(t\right){\left|\right|}_{X}\geq c>0$$

*for all sufficiently large n.*

*2. The linearized first-order propagator $U(t,t_{0})$ about ${\tilde{G}}_{\alpha }$ admits an exponentially growing mode on I: there exist $w_{0}\neq 0$ and λ>0 with*

(85) $$||U\left(t,t_{0}\right)w_{0}{\left|\right|}_{X}≳e^{\lambda (t-t_{0})}\left|\right|w_{0}\left|\right|\;\mathrm{for}\;t\in I$$

*When either condition holds, we say ${\tilde{G}}_{\alpha }$ is locally unstable on the patch $V_{\alpha }$ (or simply unstable on I).*

To obtain a complete proof, the following technical difficulties still need to be resolved: (1) Well-posedness of the coupled system, i.e., one has to prove local (and preferably global) existence, uniqueness, and continuous dependence for the metric Vlasov system. This is nontrivial because the PDEs are nonlinear hyperbolic coupled to kinetic transport. (2) Spectral comparison, i.e., showing that the linearized dispersion relations converge to the true Lyapunov exponents of the Hamiltonian flow requiring a rigorous spectral mapping between the kinetic linearization and finite-dimensional variational equations. (3) Control of moments, i.e., passing from pointwise $R_{i}^{j}$ diagnostics to moment-averaged source terms needs uniform bounds on high-order moments and propagation of regularity for *f*. (4) Energy estimates across scales, i.e., one must obtain energy (dispersion) estimates that control the back reaction loop without losing derivatives.

**Conjecture** **2.** Global stability for dissipative hyperbolic geometric flows: Suppose a finite covering of H(t) by patches $\left\{V_{\alpha }\right\}$ is given, and on each patch, the hypotheses (R1)–(R4) hold uniformly with the same constants $C_{L},\mu ,C_{N},$ and the patch reconstructions x(y) and F match on overlaps up to small residuals δ. Then there exists $\epsilon_{*}\left(\delta \right)>0$ such that any global initial perturbation with ${\left||w\left(0\right)|\right|}_{X_{global}}\leq \epsilon \leq \epsilon_{*}\left(\delta \right)$ yields a unique global solution G(t) and exponential decay to the reference family on each patch; in the limit δ→0, the global decay constant is uniform and the ungauged metrics converge exponentially to the geometric limit determined by the reference solution.

The following technical difficulties remain to be resolved in order to prove the conjecture: (1) Spectral instability to nonlinear front formation, i.e., proving that a localized unstable seed produces a coherent traveling front requires control of the nonlinear evolution of unstable modes and justification of the linear prediction beyond short times. (2) Selection of dominant k-modes, i.e., rigorous identification of the most unstable wavenumber and the resulting envelope dynamics (group velocity, growth) needs spectral gap estimates and possibly Evans function or modulational stability analysis. (3) Interaction with kinetic dispersion, i.e., the kinetic transport can smear or detune resonances. Quantifying this effect requires dispersive estimates for the Vlasov part and coupling terms.

The following non-dissipative hyperbolic geometric flow conjectures replace exponential decay by coercivity modulo neutral symmetry modes. This approach isolates and controls the genuinely dynamical degrees of freedom (keeping the physically meaningful dynamics under control) while allowing “harmless” motion along symmetry directions (infinitesimal diffeomorphisms and reparametrizations). More explicitly, we suggest the following framework: (i) fix a gauge to remove coordinate degeneracy and write the flow as a first-order quasilinear hyperbolic system in the energy space $X=H^{s}\times H^{s-1},s>\frac{n}{2}+1$; (ii) identify the finite-dimensional neutral subspace *N* generated by symmetry modes and project perturbations onto $N^{⊥}$; (iii) construct a quadratic energy *E* for the linearized problem and verify coercivity on $N^{⊥}$; and (iv) use modulation to control slow drift along *N* while closing a bootstrap argument via energy estimates to show that the orthogonal component to *N* remains uniformly O(ϵ). Under the stated spectral and nonlinear smallness assumptions, we conjecture that this may imply orbital stability: small initial perturbations remain for all time in a small tubular neighborhood of the symmetry orbit of the reference trajectory (the orthogonal part is uniformly controlled and the neutral coordinates vary only by O(ϵ)). Since the system is conservative (no damping), we do not claim exponential convergence; rather, upgrading to asymptotic stability would require additional dispersive or radiation estimates or an explicit damping mechanism [10,11].

**Conjecture** **3.** *Local stability and instability for non-dissipative hyperbolic geometric flows: Fix a patch $V_{\alpha }$ and a reference solution ${\tilde{G}}_{\alpha }\left(t\right)$ on an interval I (or for t≥0). Assume*

***(S1) Regularity and well-posedness.*** *${\tilde{G}}_{\alpha }\in C^{2}\left(I;H^{s}\right)$ and the gauged perturbation equation is well-posed in X with continuous dependence on initial data.*

***(S2) No unstable spectrum.*** *The frozen-time linearized operators have no eigenvalues with a positive real part; the linear propagator $U_{\alpha }\left(t,s\right)$ is uniformly bounded (at most polynomial growths) on X for t,s∈I.*

***(S3) Coercivity modulo symmetries.*** *There exists a finite-dimensional neutral subspace $N_{\alpha }\subset X$ generated by infinitesimal diffeomorphisms and reparametrizations and a quadratic energy $E_{\alpha }$ for the linearized problem which is coercive on $N_{\alpha }^{⊥}$:*

(86) $$c_{1}\left|\right|w_{⊥}{\left|\right|}_{X}^{2}\leq E_{\alpha }\left(w_{⊥}\right)\leq c_{2}\left|\right|w_{⊥}{\left|\right|}_{X}^{2}\;\mathrm{for}\;w_{⊥}⊥N_{\alpha }$$

***(S4) Quadratic nonlinearity.*** *The nonlinear remainder is quadratically small as in (R4).*

Then there exists $\epsilon_{*}>0$ such that for any initial perturbation $w_{\alpha }\left(0\right)$ with $\left|\right|w_{\alpha }\left(0\right)\left|\right|\leq \epsilon \leq \epsilon_{*}$, the solution decomposes uniquely as

(87) $$w_{\alpha }\left(t\right)=\Pi_{N_{\alpha }}w_{\alpha }\left(t\right)+w_{\alpha ,⊥}\left(t\right)$$

*where $\Pi_{N_{\alpha }}$ is the projection onto the neutral subspace and $w_{\alpha ,⊥}$ is orthogonal to $N_{\alpha }$. There exists C>0 with*

(88) $$\left|\right|w_{\alpha ,⊥}\left(t\right){\left|\right|}_{X}\leq C\epsilon \;for\;t\in I$$

*and the neutral coordinates remain O(ϵ). Thus, ${\tilde{G}}_{\alpha }$ is orbitally (Lyapunov) stable on the patch $V_{\alpha }$. If the frozen linearization on the patch admits an exponentially growing eigenmode, then ${\tilde{G}}_{\alpha }$ is locally unstable on that patch.*

The following issues remain to be addressed to complete the proof: (1) Completeness and nondegeneracy, i.e., proving that no other hidden channels arise from higher moments or nonlocal phase-space structure requires uniform moment control and closure justification. (2) Rigorous mode decomposition, i.e., establishing orthogonality, spectral separation, and boundedness of coupling operators in appropriate function spaces is needed to treat mode conversion and energy transfer. (3) Gauge and constraint handling, i.e., vector fields introduce gauge freedom. Proving well-posedness and decoupling requires careful constraint analysis and choosing gauges that are compatible with the symplectic structure.

**Conjecture** **4.** *Global stability for non-dissipative hyperbolic geometric flows: If a finite covering $\left\{V_{\alpha }\right\}$ and a family of reference short solutions $\left\{{\tilde{G}}_{\alpha }\right\}$ satisfy (S1)–(S4) uniformly, and overlap residuals are small, then small global initial perturbations remain for all time in a small tubular neighborhood of the union of symmetry orbits of the reference family.*

The following technical obstacles to a full proof should be addressed: (1) Error accumulation and propagation, i.e., showing that local reconstruction errors do not amplify through hyperbolic propagation or feedback with the kinetic moments requires uniform stability estimates for the numerical/analytic patch coupling. (2) Adaptive refinement control, i.e., proving that a finite refinement strategy suffices to keep errors below thresholds over long times is difficult without quantitative a priori estimates on solution regularity. (3) Coupling with Vlasov sampling noise; i.e., particle sampling or discretization of *f* introduces stochastic errors. Controlling their effect on the continuous PDE requires probabilistic or deterministic sampling error bounds.

## 4. Schematic Hénon–Heiles Example

We take for a simple illustration here the Hénon–Heiles model. The model and patches:

(89) $$\begin{array}{c} V\left(x,y\right)=\frac{1}{2}\left(x^{2}+y^{2}\right)+\lambda \left(x^{2}y-\frac{1}{3}y^{3}\right)\\ with\;\lambda =1,\Phi \left(x,y\right)=\frac{E}{E-V(x,y)},E=1.5 \end{array}$$

Let

(90) $$\begin{array}{c} U_{1}=\left\{x^{2}+y^{2}<r_{1}^{2}\right\},r_{1}=0.25\\ U_{2}=\left\{{\left(x=0.15\right)}^{2}+{\left(y-0.05\right)}^{2}<r_{2}^{2}\right\},r_{2}=0.25 \end{array}$$

and the overlap be $U_{12}=U_{1}\cap U_{2}$.

Next, apply the Poisson–Hodge componentwise problem (statement). On each patch, solve for a=1,2

(91) $$\begin{array}{c} \Delta_{x}\phi_{i,a}\left(x\right)=-{▽}_{x}\cdot \left(\Phi^{-1}\left(x\right)X_{i,a}^{sample}\left(x\right)\right)\;in\;U_{i}\\ \phi_{i,a}{|}_{\partial U_{i}}=g_{i,a} \end{array}$$

with the sampling model $X^{sample}\left(x\right)=x$ and $g_{i,a}\left(x\right)=x_{a}$ on $\partial U_{i}$.

Apply the small patch closed-form (constant $\Phi \approx \Phi_{0}$) approximation. Then $\Delta \phi_{a}=-\Phi_{0}^{-1}$ and on a disk centered at *c*

(92) $$\begin{array}{c} \phi_{a}\left(x\right)={\left(x-c\right)}_{a}-\frac{\Phi_{0}^{-1}}{4}||x-{c||}^{2},a=1,2 \end{array}$$

so on $U_{1}$ (center 0) $\phi^{\left(1\right)}\left(x\right)=(x-\frac{a}{4}{\left||x|\right|}^{2},y-\frac{a}{4}{\left||x|\right|}^{2})$ with $a=\Phi_{0}^{-1}$.

Next, compute first-order transition on $U_{12}$. With $y=\phi^{\left(1\right)}\left(x\right)$ and c=(0.15,0.05), to O(a)

(93) $$\begin{array}{c} T_{12}\left(y\right)=\phi^{\left(2\right)}\circ {\left(\phi^{\left(1\right)}\right)}^{-1}\left(y\right)=y-c+\frac{a}{4}{\left(2y\cdot c-|c\right|}^{2})1+O\left(a^{2}\right) \end{array}$$

where $1={\left(1,1\right)}^{T}$. The Jacobian is $DT_{12}=I+\frac{a}{2}c⊗1+O\left(a^{2}\right)$.

Derive the metric and curvature residual (leading order). The metric pullback error is

(94) $$\begin{array}{c} \Delta G\left(y\right)=\Phi \left(T_{12}\left(y\right)\right)DT_{12}^{T}DT_{12}-\Phi \left(y\right)I\approx \Phi \left(y\right)\left(M+M^{T}\right)+\left(▿\Phi \cdot \left(T_{12}-y\right)\right)I \end{array}$$

with $M=DT_{12}-I=O\left(a\right)$. For E=1.5, one has $a\approx \frac{2}{3}$ and the transition/Jacobian deviations are $O(10^{-2})$ for these patch sizes. These feed into the curvature bound

(95) $$\left|\right|R^{res}{\left|\right|}_{L^{\infty }}\leq K_{1}\epsilon_{exact}+K_{2}\epsilon_{diam}+K_{3}\epsilon_{ortho}+K_{4}\epsilon_{interp}$$

where the computed $\left|\right|T_{12}-Id\left|\right|$ and $||DT_{12}-I\left|\right|$ feed into $\epsilon_{ortho,i}$ and $\epsilon_{exact,i}$.

Use the closed-form $\phi_{i}$ as a first approximation, compute sample pairs $\left(\phi^{\left(1\right)}\left(x\right),\phi^{\left(2\right)}\left(x\right)\right)$ on $U_{12}$, evaluate $T_{12}$ and $DT_{12}$ numerically, and feed $\left|\right|T_{12}-Id{\left|\right|}_{C^{1}}$ and ${\left||\Delta G|\right|}_{C^{2}}$ into the residual estimate above. Decide whether to refine patches (reduce radii) or increase sampling density to reduce $\epsilon_{interp}$ and $\epsilon_{exact}$.

## 5. General Remarks on Eisenhart–Duval Lift Compared with the Hamilton Manifold

Our Hamilton manifold is a patchwise, curvature-driven reconstruction whose geometry is defined to reproduce the separation of Newton trajectories on configuration space. Starting from the second-order geodesic deviation equation for the conformal (Jacobi) metric, we obtain an orbit deviation equation for the time-independent potential Hamiltonian model (constant energy). The dynamical curvature tensor $R_{qmn}^{l}$ that appears in this equation is the quantity we reconstruct and control via residuals. Unlike introducing a metric (M,g) and a corresponding curvature, $R_{qmn}^{l}$ is the tensor that actually governs trajectory separation in the potential Hamiltonian model. Its spectral structure determines local converging or diverging. Positive curvature directions correspond to local converging and stability, while negative eigenvalues indicate local exponential separation and instability.

The Hamilton manifold is built directly from $R_{qmn}^{l}$. Its distinctive elements are the patchwise Poisson–Hodge reconstruction and a regularized hyperbolic geometric flow for the geometric blocks. Treating *t* as a parameter means a family of spatial geometries G(t) whose evolution is an external hyperbolic PDE on the space of metrics (inertial, wave-like metric response), so physically G(y,t) models time-varying inertia or material geometry and disturbances propagate with finite speed.

The Hamilton manifold treats the metric (or other fields Φ) as genuine dynamical variables evolving by a hyperbolic geometric flow in inference time τ, and physically, this means the inertia or the geometry not only parametrizes particle motion but also carries its own wave-like dynamics and inertia, so heuristically, disturbances in *G* propagate as waves and interact back with particles.

The Hamilton manifold endows the geometric fields Φ, with genuine wave-like dynamics, and wave propagation stability. Since the Hamilton manifold also retains the phase space (symplectic geodesic flow) structure with a conserved energy and a well-posed linearization (i.e., paired eigenvalues and Krein signatures), Lyapunov, spectral, and KAM tools become applicable to obtain coercivity, modal bounds, and long time stability. Also, finite-speed propagation for field perturbations results in local disturbances which cannot instantaneously “destabilize” distant regions, so it is possible to design absorbing boundary conditions and robust control of metric perturbations by promoting *G* to a hyperbolic dynamical field.

This combined analysis, where it becomes possible to linearize the coupled particle-field system and use spectral analysis for particle modes and energy estimates for field modes, gives stronger and more complete stability statements.

The Eisenhart–Duval (ED) manifold [12] is a kinematic geometric encoding, i.e., it embeds the mechanical system on configuration space into an (*n* + 2)-dimensional Lorentzian spacetime $\left(\tilde{M},\tilde{g}\right)$, so that solutions of the Newton (Hamilton) equations correspond to the projection of a null geodesic of $\tilde{g}$. This makes time t a coordinate on the spacetime, while introducing an extra null coordinate u, and packages the kinetic metric and the potential V (*x*) into a single Lorentzian metric. It restores covariance and causal cones for particle motion, and along with the geodesic variational principle reproduces the original dynamics.

However, the Eisenhart–Duval manifold does not encode a dynamical field theory for the metric (it does not by itself make the metric/inertia or potential into dynamical wave fields), so intrinsic energy estimates for the geometry itself are not available. As so, the Eisenhart–Duval geometric embedding is weaker for stability analysis than the Hamilton manifold since it replaces an autonomous, symplectic phase-space structure with a kinematic spacetime picture that lacks the conserved energy and symplectic spectral structure needed for modal, KAM, and long time orbital stability arguments. The ED embedding can therefore obscure spectral pairings, signatures, and the Lyapunov functionals that the Hamilton manifold makes available.

Time-dependent potential Hamiltonian systems [13]

(96) $$H_{p}=\frac{1}{2m}p_{i}p_{j}\delta^{ij}+V\left(y,t\right)$$

may be addressed by two principled geometric strategies. One is the Eisenhart–Duval (ED) lift, which embeds time dependence into a higher-dimensional Lorentzian geometry.

The other is to follow the Hamilton manifold construction, while treating

(97) $$G_{target}\left(y,t\right):=\frac{E(y,t)}{E(y,t)-V(y,t)}$$

as an instantaneous target and design dynamics that relax $G_{\alpha ,ab}$ toward it.

The second approach is the use of the Hamilton manifold with a time-dependent conformal metric and instantaneous Jacobi metric target; then, spatial paths approximate geodesics of $G_{J}^{\alpha }\left(y,t\right)$

(98) $${G_{J}}_{ij}\left(y,t\right):=\frac{E_{p}}{E_{p}-V\left(y,t\right)}\delta_{ij}{|}_{\alpha }$$

where $E_{p}=E_{p}\left(t\right)$ or $E_{p}=E_{p}\left(y,t\right)$, defined by

(99) $$E_{p}^{\alpha }\left(t\right)=\frac{\int_{\alpha }H_{p}\left(y,p,t\right)f_{\alpha }\left(y,p,t\right)dpdx}{\int_{\alpha }f_{\alpha }\left(y,p,t\right)dpdx},\;E_{p}^{\alpha }\left(y,t\right)=\int_{\alpha }H_{p}\left(y,p,t\right)f_{\alpha }\left(y,p,t\right)dp$$

where $H_{\alpha ,p}\left(y,p,t\right)=\frac{1}{2m}p_{i}p_{j}\delta^{ij}+V\left(y,t\right)$, so that $E_{p}^{\alpha }\left(t\right)$ is the mean particle energy.

Let $f_{\alpha }\left(t\right)$ be time-dependent and satisfy the Vlasov equation

(100) $$\partial_{t}f_{\alpha }=-L_{X_{H_{\alpha ,G}}}f_{\alpha }$$

embedding two Hamiltonians and one phase-space density $f_{\alpha }$ in $E_{p}^{\alpha }$. So metric $G_{\alpha ,ab}$, distribution $f_{\alpha }$, and $E_{p}^{\alpha }$ evolve self-consistently.

Particles move according to the geometry defined by $G_{\alpha }$ governed by $H_{\alpha ,G}$, while $H_{\alpha ,p}$ determines the energy.

Define the following moments

(101) $$\rho \left(y,t\right)=\int_{\alpha }f_{\alpha }dp\;,P_{i}\left(y,t\right)=\int_{\alpha }p_{i}f_{\alpha }dp\;,M_{\alpha ,ij}\left(y,t\right)=\int_{\alpha }p_{i}p_{j}f_{\alpha }dp$$

then, the local energy density results in

(102) $$E_{p}^{\alpha }\left(y,t\right)=\int_{\alpha }H_{p}\left(y,p,t\right)f_{\alpha }\left(y,p,t\right)dp=\frac{1}{2m}\delta^{ij}M_{ij}+\rho V$$

Differentiate and use the Vlasov equation

(103) $$\partial_{t}E_{p}^{\alpha }\left(y,t\right)=\int_{\alpha }f_{\alpha }\left\{H_{\alpha ,G},H_{\alpha ,p}\right\}dp+\int_{\alpha }f_{\alpha }\partial_{t}H_{\alpha ,p}dp$$

Therefore,

(104) $$\partial_{t}E_{p}^{\alpha }\left(y,t\right)=\rho \partial_{t}V+\frac{1}{m}\partial_{y^{k}}Vg^{kl}P_{l}-\frac{1}{2m^{2}}\partial_{y^{k}}g^{ij}T_{ij}^{k}$$

with the third-order moment

(105) $$T_{\alpha ,ij}^{k}\left(y,t\right)=\int_{\alpha }p^{k}p_{i}p_{j}f_{\alpha }dp$$

The first term in $\partial_{t}E_{p}^{\alpha }\left(y,t\right)$ is the $\rho \partial_{t}V$ energy change from explicit time-dependence of the potential. The second term is $\frac{1}{m}\partial_{y^{k}}Vg^{kl}P_{l}$, reflecting the advection of the potential by the metric-dependent particle velocity. It is a transport coupling between the potential gradient and the momentum measured with the metric *g*. The third term is $-\frac{1}{2m^{2}}\partial_{y^{k}}g^{ij}T_{ij}^{k}$, which is a third-moment (non-isotropic) coupling, so anisotropy in momentum space and metric gradients produce local gains or losses of the $H_{\alpha ,p}$ energy density. A tight nonlinear feedback loop that can amplify or damp the kinetic structure depending on the sign and anisotropy.

Geometrically, it reflects the key geometric source; i.e., spatial variations in the metric do work on the particle ensemble. The extra term is the work of metric inhomogeneity on the kinetic distribution, a geometric generalization of “strain work”, where spatial changes of the metric redistribute kinetic energy measured in a fixed reference.

An equivalent flux form of $\partial_{t}E_{p}^{\alpha }\left(y,t\right)$ may be represented by grouping the transport pieces into a divergence of an energy flux plus explicit sources.

The energy flux built with $H_{\alpha ,p}$

(106) $$F^{k}:=\int_{\alpha }H_{\alpha ,p}\frac{p^{k}}{m}f_{\alpha }dp$$

Then, after algebra and integration by parts in *y*, one obtains

(107) $$\partial_{t}E_{p}^{\alpha }\left(y,t\right)+\partial_{y^{k}}F^{k}=\rho \partial_{t}V-\frac{1}{2m^{2}}\partial_{y^{k}}g^{ij}T_{ij}^{k}+S_{mom/time}\left(\mathrm{terms}\;\mathrm{that}\;\mathrm{can}\;\mathrm{be}\;\mathrm{absorbed}\;\mathrm{into}\partial_{y^{k}}F^{k}\right)$$

so the “non-conservative source” is the metric gradient coupling to the third-order moment, while the $\rho \partial_{t}V$ term remains the explicit Hamiltonian time-dependence source.

The $S_{mom/time}$ term denotes terms that arise from $-\frac{V}{m}g^{kl}\partial_{y^{k}}P_{l}$ after using the momentum equation. Those terms are either expressible as additional divergences of known fluxes (hence, absorbable into $\partial_{y^{k}}F^{k}$) or time-derivative terms of lower moments (which may be paired with geometric energy/time terms when forming the total conserved energy of the coupled system).

In the conformal limit, the third-order term vanishes and the usual flat space balance is recovered

(108) $$\partial_{t}E+▽\cdot F=\rho \partial_{t}V$$

The flux representation reflects how energy flows between “spatial cells”, i.e., the remaining non-divergence terms may be considered as sources or sinks that are balanced by field or “geometric” energy if total energy is to be conserved.

Next, one may consider the ensemble (moment) preservation

(109) $$\int_{\alpha }f_{\alpha }\left(\left\{H_{p},H_{\alpha ,G}\right\}+\partial_{t}H_{p}\right)dp=0$$

which is a finite set of constraints on moments (i.e., ρ,P,T). It attempts to align the two Hamiltonians at the level of the measured energy $E_{p}^{\alpha }$.

Therefore, defining the residual

(110) $$R^{\alpha }\left(y,t\right):=\int_{\alpha }f_{\alpha }\left(\left\{H_{p},H_{\alpha ,G}\right\}+\partial_{t}H_{p}\right)dp$$

measures how well the ensemble preserves the $H_{\alpha ,p}$ “shell”.

Another approach may be the use of the Eisenhart–Duval (ED) lift of the time-dependent Hamilton manifold discussed above, producing a single lifted metric $\tilde{g}$

(111) $${\tilde{g}}_{\alpha }=G_{\alpha ,ij}\left(q,t\right)dq^{i}dq^{j}+2dtdu-2V_{\alpha }\left(q,t\right)dt^{2}$$

with lifted coordinates $x^{a}=\left(u,t,q^{i}\right)$. The $\left(G_{\alpha },V_{\alpha }\right)$ are literal metric blocks of ${\tilde{g}}_{\alpha }$ and thus appear as geometric fields in the ED picture.

The Hamiltonian on the lift

(112) $$H_{\alpha ,ED}\left(y,p\right)=\frac{1}{2m}{\tilde{g}}^{\alpha ,ab}\left(x\left(y\right)\right)p_{a}p_{b}$$

Then, the Eisenhart–Duval (ED) lift embeds those fields into a Lorentzian metric, whose spatial projection reproduces the geometric evolution for $G_{\alpha }$.

Taking phase-space moments of the double bracket against a distribution $f_{\alpha }\left(t\right)$ (Vlasov) yields

(113) $$\int_{\alpha }\left\{H_{\alpha ,ED},\left\{H_{\alpha ,ED},{\tilde{g}}_{\alpha }\right\}\right\}f_{\alpha }dp$$

which produces tensorial data moments that act as source terms in the ED lift of the Hamilton manifold.

The pair $\left(G_{\alpha },V_{\alpha }\right)$ becomes blocks of a Lorentzian metric ${\tilde{g}}_{\alpha }$, and null geodesics of ${\tilde{g}}_{\alpha }$ project to particle trajectories. In this approach, kinetic moments appear as tensorial geometric sources (Lie derivatives, curvature-like objects) for ${\tilde{g}}_{\alpha }$’s evolution, where metric waves, curvature, and causal structure are intrinsic geometric objects. In the ED lift representation, one may derive, from a single action, the Noether currents and the total conserved energy momentum. This helps ensure consistency when metric inertia is included (the total energy of the coupled system of “particles + metric” may be a robust conserved quantity in a variational setting). Geometry becomes a dynamical field with its own wave physics.

Heuristically, particles create “ripples” in a single spacetime fabric, while those ripples propagate with light cone causality and then refract particle geodesics.

In the Hamilton manifold representation (phase space), particle motion is Hamiltonian flow while metric waves (driven fields) live on configuration space, and geometry is an external field that modifies the flow (alter particle characteristics).

The two approaches are formally equivalent as “encodings” of the same dynamics, but they differ in what they make explicit, and the coupling of particles to geometry.

Two complementary geometrization routes appear in the recent literature. Paliathanasis’s [14] approach addresses the Jacobi with the Eisenhart combination to give a general symmetry count criterion (i.e., maximal Jacobi symmetry ↔ linearizability), while Paliathanasis’s study in [15] constructs Eisenhart lifts for GR minisuperspaces, suggesting that the extended metrics are conformally flat so the field equations reduce to linear free particle form. However, attempting to generalize that mechanism from finite minisuperspace constructions to the Hamilton manifold requires replacing finite symmetry counting by a functional analytic treatment of the configuration-space metric, along with careful attention of gauge and boundary data. Explicit tests on truncated field models are required to probe whether a functional Jacobi–Eisenhart reduction can produce an analogous linearization.

## 6. Conclusions

We have presented a patchwise geometric construction that embeds a broad class of potential model Hamiltonian dynamics into a local Riemannian representation on the Hamilton patches. We used this construction to formulate a hyperbolic geometric flow for the pulled metrics and introduce a representation with (i) explicit frame-adapted reconstruction and Poisson–Hodge solvability conditions that produce Hamilton coordinates on each patch; (ii) uniform quantitative control of connection and curvature residuals in terms of interpolation, exactness, and Jacobian tolerances; and (iii) short time well-posedness for the quasilinear hyperbolic metric evolution coupled to a kinetic closure.

Our representation makes it possible to associate local curvature diagnostics (the dynamical curvature tensor) with local stability of trajectories and to suggest patchwise and global stability conjectures for both dissipative and conservative hyperbolic geometric flows.

The convergence stability conjectures we propose address two complementary regimes. In the dissipative setting, a spectral gap for the gauged linearization implies exponential convergence of small perturbations, while in the conservative setting, exponential decay is replaced by coercivity of a quadratic energy on the orthogonal complement of a finite-dimensional symmetry subspace. This coercivity, as we suggest, implies orbital (Lyapunov) stability on each patch. Furthermore, we propose that if the coercivity constants are uniform across patches, and overlap residuals are sufficiently small, then global orbital stability of the reconstructed Hamilton manifold is implied. In both cases, the same linear instability criteria is suggested; the presence of an exponentially growing eigenmode on a patch (for a fixed time) provides a local criterion for instability.

Our framework may suggest practical diagnostics for detecting the onset of chaotic behavior in Hamiltonian systems by exploring whether local instabilities can propagate globally.

In summary, the Hamilton patch reconstruction and the associated Hamilton manifold with atlas-dependent time and hyperbolic geometric flow provide a geometric framework construction that may imply a new approach for studying stability and instability in Hamiltonian mechanics. We believe that our work may bridge classical curvature-based diagnostics and PDE stability theory and may admit a new pathway to rigorous verification and numerical exploration of stability properties in a wide class of mechanical systems.

## Data Availability

The original contributions presented in this study are included in the article. Further inquiries can be directed to the corresponding authors.
